# Trehalose-Rich, Degradable Hydrogels Designed for Trehalose Release under Physiologically Relevant Conditions

**DOI:** 10.3390/polym11122027

**Published:** 2019-12-06

**Authors:** Małgorzata Burek, Ilona Wandzik

**Affiliations:** 1 Department of Organic Chemistry, Bioorganic Chemistry and Biotechnology, Faculty of Chemistry, Silesian University of Technology, B. Krzywoustego 4, Gliwice 44-100, Poland; malgorzata.burek@polsl.pl; 2 Biotechnology Center, Silesian University of Technology, B. Krzywoustego 8, Gliwice 44-100, Poland

**Keywords:** trehalose, hydrogel, glycopolymer, hydrolytic degradation, trehalose delivery

## Abstract

Trehalose, a natural disaccharide, is primarily known for its ability to protect proteins from inactivation and denaturation caused by a variety of stress conditions. Furthermore, over the past few years, it has emerged as a promising therapeutic candidate for treatment of neurodegenerative diseases. Herein, we examine the attachment of trehalose to polymers for release under selected physiologically relevant conditions. The proposed strategies are evaluated specifically using hydrogels undergoing simultaneous degradation during trehalose release. These materials are fabricated via copolymerization of the appropriate acrylamide-type monomers with polymerizable trehalose esters or benzylidene acetals. This provides trehalose release in a slightly alkaline (i.e., pH 7.4) or mildly acidic (i.e., pH 5.0) environment, respectively. Using this method materials containing up to 51.7 wt% of trehalose are obtained. The presented results provide a solid basis for future studies on polymeric materials intended for trehalose release in biological systems.

## 1. Introduction

Trehalose, a natural disaccharide, is commonly found in various organisms and primarily known for its ability to protect proteins from inactivation or denaturation caused by an array of stress conditions. Its unique properties allows trehalose to be employed as a stabilizer in some pharmaceutical protein/polypeptide formulations [1]. Recently, numerous studies have shown that trehalose acts as an autophagy modulator, making it applicable as a prophylactic drug for several diseases in which autophagy plays an important role [2,3,4]. Autophagy is a lysosome-dependent mechanism for intracellular degradation, which enables cells to self-degrade intracellular components within lysosomes for recycling. Dysregulation of autophagy is associated with multiple human diseases. Recent studies have focused on autophagy stimulation by trehalose and its therapeutic impact on different diseases, especially neurodegenerative diseases, has been extensively studied [3,4]. Reports have shown that in vitro studies were carried out on cellular models of PD [5,6], HD [7] and in vivo studies on animal models of AD [8] and ALS [9]. Furthermore, trehalose-induced autophagy can function as both a proviral and antiviral depending on the virus and context. Belzite et al. [10] demonstrated autophagy-inducing activity and inhibitory effects against human cytomegalovirus in multiple cell types. DeBosch et al. [11] showed that trehalose could be implemented in the treatment of nonalcoholic fatty liver disease. Furthermore, a recent search of ClinicalTrials.gov shows two clinical trials involving trehalose as an autophagy modulator. In the first study trehalose’s ability to induce macrophage autophagy-lysosomal biogenesis as a therapy for atherosclerosis has been the groundwork [12]. The use of intravenous trehalose to reduce vascular inflammation in acute coronary syndrome is currently in phase two clinical trials. The second study examines bipolar disorder where trehalose is in phase three clinical trials to assess its efficacy and tolerability of trehalose as adjunctive treatment to lithium. The basis of the study is the assumption that enhanced autophagy may be involved in the therapeutic action of antidepressant and mood-stabilizing drugs.

The aforementioned studies of autophagy-modulation by trehalose makes trehalose promising for potential biological and practical applications. Therefore, materials, especially nanoparticles, that release trehalose at physiologically relevant conditions could be an alternative for simple administration of trehalose in classical formulations. Such materials have the advantage of reduced trehalose clirens, as well as extended stability by protecting it from rapid enzymatic hydrolysis into glucose by trehalase.

**Figure 1:**
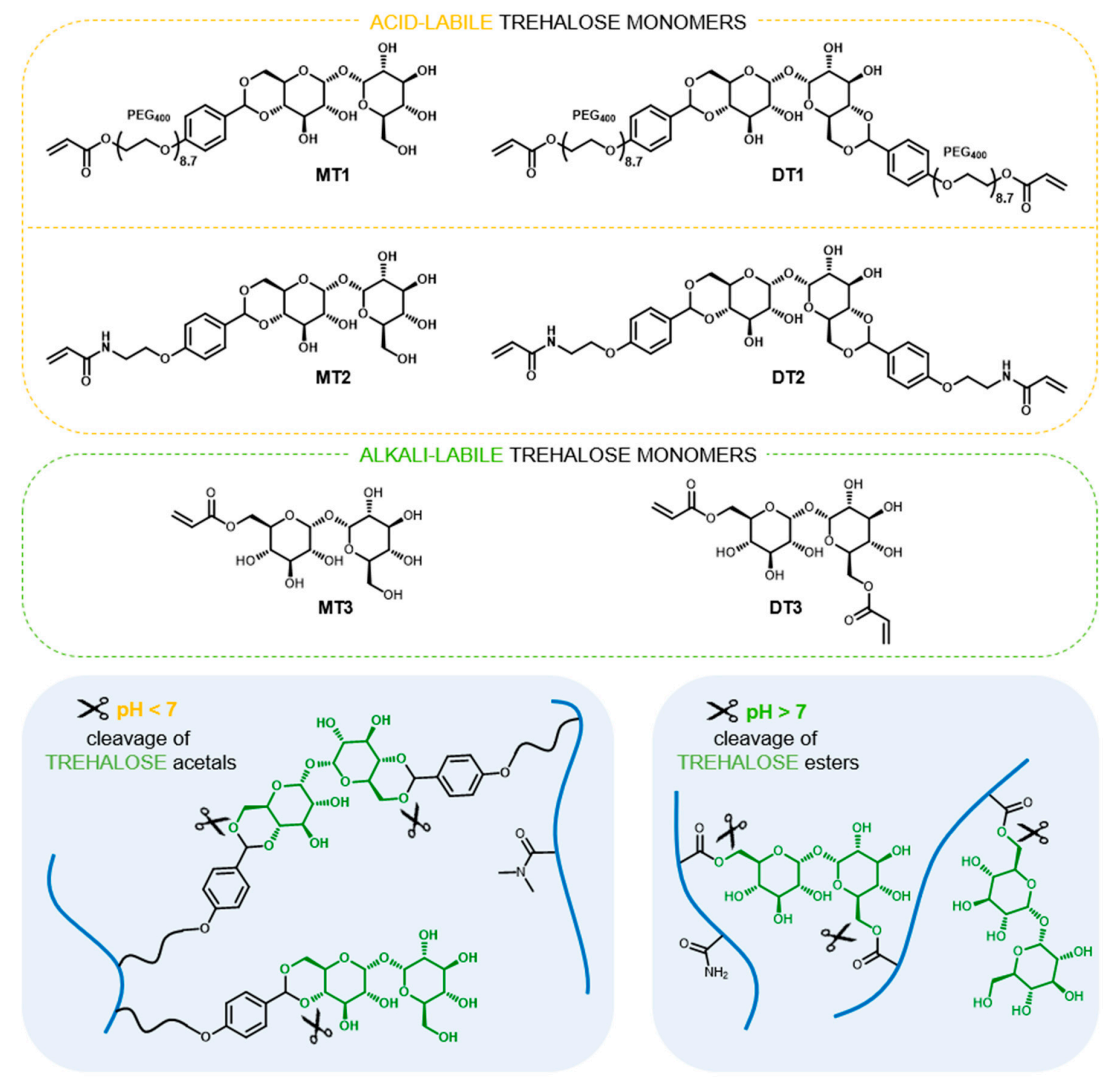
Structures of trehalose monomers and schematic presentation of polymer network of trehalose-rich, degradable hydrogels.

In 1979, Kurita et al. [13] presented the synthesis of trehalose containing polymer for the first time. Since this, covalent incorporation of trehalose in macromolecules has received continuous interest with more than fifty known publications available (Scopus database). Polymers containing trehalose were obtained using various polymerization and post-polymerization techniques and were studied as e.g., excipients for protein stabilization under deactivating conditions [14,15,16,17], nonviral nucleic acid carriers [18,19,20], thermogelling hydrogel matrices for 3D cancer cell culture [21], and magnetic nanoparticles for selective interactions with mycobacteria [22]. In our recent studies, we presented the utilization of trehalose derivatives in hydrogels synthesis as hydrolytically-labile crosslinkers [23,24,25,26,27]. We have shown that appropriate structure design of trehalose crosslinker and careful comonomer selection enables modulation of degradation rate and fabricate hydrogels that degrade at physiological pH [25,26]. To the best of our knowledge, the release of substantial amounts of trehalose under physiologically relevant conditions has yet to be developed. Considering trehalose’s high therapeutic potential and lack of approaches towards synthesis of trehalose releasing polymers, herein, we investigate trehalose-rich materials capable of hydrolytic release of trehalose at physiological pH. The proposed approach is based on trehalose benzylidene acetals or esters as monomers for free radical polymerization, and is evaluated specifically using hydrogels, undergoing simultaneous degradation during trehalose release. To elaborate on this proposal various pairs of trehalose monomers were designed (Figure 1). Each pair consists of a trehalose derivative with two polymerizable sites that act as a hydrolytically-labile crosslinker and trehalose derivative with one polymerizable site allowing the introduction of trehalose into the polymer network as a pending moiety. The sets of trehalose mono- and dibenzylidene acetals MT1/DT1 and MT2/DT2, respectively, were used to promote trehalose release under mildly acidic conditions, which characterize tumor sites or inflammatory tissues as well as endosomes and liposomes. In turn, the pair of trehalose mono- and diacrylate MT3/DT3 was selected in order to obtain degradable hydrogels under slightly alkaline conditions (pH 7.4) that normally prevails in the human body. Based on the obtained results of our previous study [25], the main comonomer for hydrogel formation was carefully selected from the acrylamide-type monomer family.

## 2. Experimental 

### 2.1. Materials and General Methods

Acrylamide (AM), acryloyl chloride, amberlyst-15, ammonia aqueous, ammonium persulphate (APS), 2,2’-Azobis[2-(2-imidazolin-2-yl)propane] dihydrochloride (V-044), Celite 501, hydrochloric acid, hydroquinone, *p*-hydroxybenzaldehyde, lithium tetrafluoroborate, methanesulfonyl chloride, phosphate buffered saline (PBS) tablets, polyethylene glycol (PEG_400_), pyridine, silver(I) oxide (Ag_2_O), sodium hydroxide, *N,N,N’,N’*-tetramethylethylenediamine (TMEDA), *p*-toluenesulfonic acid, tosyl chloride, anhydrous trehalose, triethylamine (TEA), and trimethyl orthoformate, trimethylsilyl chloride were purchased from Sigma Aldrich (Steinheim, Germany or Saint Louis, Missouri, US), Acros Organics (Geel, Belgium) or TCI (Tokyo, Japan) and used directly without any purification. *N,N*-dimethylacrylamide (DMAM) (Sigma Aldrich, Steinheim, Germany or Saint Louis, Missouri, US) were purified by passing through a column filled with basic aluminum oxide to remove inhibitor. Anhydrous solvents were purchased from Acros Organics (Geel, Belgium) and stored over molecular sieves under inert atmosphere. All other solvents and inorganic salts were purchased from Avantor Performance Materials Poland S.A. (Gliwice, Poland). Trehalose Assay Kit was purchased from Megazyme (Bray, Ireland).

Purifications by flash column chromatography were performed on silica gel 60 (40–63 μm, Merck KGaA, Darmstadt, Germany) using automated system Isolera (Biotage, Uppsala, Sweden). Freeze-drying was carried out under 0.035 mbar at −50 °C (ALPHA 1-2 LDplus, CHRIST). Mass spectra were recorded using Electrospray Ionisation Mass Spectrometry QTRAP 4000 (AB Sciex, Foster City, California, US). NMR spectra were recorded in deuterated solvents (Deutero GmbH, Kastellaun, Germany) with internal standards using NMR spectrometer operating at 400 or 600 MHz (Varian Inc., Palo Alto, California, US).

### 2.2. Synthesis and Characterization of Trehalose Monomers

The details on MT1/DT1, MT2/DT2, MT3/DT3 monomer’s synthesis and characterization are provided in Supporting Information.

### 2.3. Synthesis and Study on Hydrogels

#### 2.3.1. Synthesis of Acid-Labile Hydrogels

Acid-labile hydrogels were synthesized using DMAM and trehalose monomers set MT1/DT1 or MT2/DT2 by thermally-initiated free-radical polymerization. The detailed feed compositions and sample codes are given in Table S1. In all entries, molar concentration of crosslinker and initiator was kept constant. Briefly, 100 µL of V-044 aqueous solution (1 M, 0.1 mmol) and the solution of crosslinker in DMAM were added to the solution of trehalose monoacetal in 1250 µL of 10 mM phosphate buffer (pH 7.5). The resulting solution was argon-purged for 15 min. and divided between five 8.5 mm diameter wells on self-made PDMS form. Polymerization was run at 45 °C for 1 h. Disc-shaped hydrogels were transferred into 10 mM phosphate buffer (pH 7.5) for purification (buffer was replaced every 12 h for 3 days). The last washing was performed in DI water. Hydrogels were air-dried for one week at room temperature and then freeze-dried.

#### 2.3.2. Synthesis of Alkali-Labile Hydrogels

Alkali-labile hydrogels were synthesized using AM and trehalose monomers set MT3/DT3 by redox-initiated free-radical polymerization at 25 °C. The detailed feed compositions and sample codes are given in Table S2. In all entries concentrations of crosslinker and initiating system were kept constant. Briefly, monomers was dissolved in 1000 µL of DI water, followed by the addition of 25 µL of TMEDA aqueous solution (912 mM, 0.023 mmol). The resulting solution was argon-purged for 15 min. and divided between four 8.5 mm diameter wells on self-made PDMS form. The polymerization was initiated by adding 10 µL of APS aqueous solution (380 mM, 0.004 mmol) to each well and left for 3 h. Disc-shaped hydrogels were transferred into DI water for purification (DI water was replaced every 12 h for 3 days). Hydrogels were air dried for one week at room temperature and then freeze dried.

### 2.4. Hydrogels Characterization

#### 2.4.1. Determination of Trehalose Content

Trehalose content in hydrogels was determined, after it had been released into solution through acid (hydrogels containing trehalose diacetals) or alkaline (hydrogels containing trehalose acrylates) hydrolysis by immersing dry hydrogel disc in appropriate volume of 0.1 M HCl or 0.1 M NaOH solution, respectively to get final concentration of 1 mg/mL. After one night of magnetic stirring at 25 °C, 800 µL of degradation solution was withdrawn, neutralized with 200 µL of 0.4 M NaOH or 0.4 M HCl solution, and subjected to enzymatic determination of trehalose. Control solutions containing a fixed concentration of trehalose in 0.1 M HCl or NaOH, were treated the same way to confirm that it is stable under degradation conditions. Trehalose was determined enzymatically using Trehalose Assay Kit (Megazyme, Bray, Ireland) in microplate assay procedure, based on standard curve. The absorbance was recorded using the Sunrise™ microplate absorbance reader (Tecan Group Ltd., Männedorf, Switzerland). Each determination was made in triplicate. 

#### 2.4.2. Characterization of Degradation Products by ^1^H NMR Spectroscopy

To acquire ^1^H NMR spectra of degradation products, degradation solutions from the previous analysis (2.3.1.) were freeze-dried and dissolved in D_2_O.

#### 2.4.3. Trehalose Release Study

Dry hydrogel discs (~25 mg) were immersed in 10 mL of buffered saline solution at 37 °C and left under magnetic stirring (200 rpm). Release from acid-labile and alkali-labile hydrogels was followed at pH 5.0 (10 mM citrate buffer) and pH 7.4 (10 mM phosphate buffer), respectively. At predetermined time intervals samples (250 µL) were withdrawn and replaced with fresh buffer. Samples were then diluted and trehalose was determined enzymatically using Trehalose Assay Kit (Megazyme International, Bray, Ireland) in microplate assay procedure, based on standard curve.

Degradation time was estimated as time necessary for hydrogel to disintegrate into clear solution.

#### 2.4.4. Equilibrium Swelling Ratio (ESR)

To determine ESR, dried hydrogel disc (~25 mg) was immersed in 10 mL of PBS pH 7.0 at 25 °C and weighed after removing surface water every 12 h until reaching equilibrium swelling. ESR was calculated from the following equation:

(1) $$\mathrm{ESR}=\frac{W_{E}-W_{D}}{W_{D}}$$

where: W_E_ is the weight of the equilibrium swollen hydrogel and W_D_ is the weight of dry hydrogel. ESR was taken as an average value of three independent measurements.

## 3. Results and Discussion

### 3.1. Hydrogels Capable of Trehalose Release under Mildly Acidic Conditions

**Figure 2:**
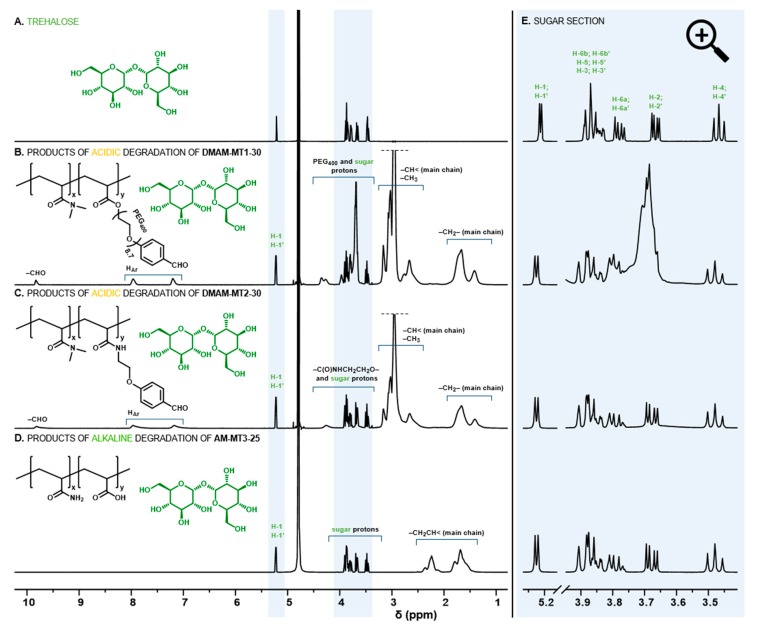
^1^H NMR spectra of trehalose (**A**) and degradation products of selected hydrogels (**B**–**D**) with magnification of the section of sugar proton signals (**E**).

The presence of the aromatic ring in benzylidene acetals of trehalose provides the possibility to modulate the rate of hydrolysis, while strongly limiting their solubility in water. Previously, we have shown that a series of trehalose benzylidene diacetal crosslinkers are soluble in aqueous polymerization solution only with the substantial addition of DMF [23,24,25,26]. Herein, we propose novel derivatives whose structures were carefully designed to increase water solubility, thus eliminating additional organic solvents. The introduction of a polyethylenoxy chain enables significant reduction of required DMF [25], therefore, we examined elongation of the polyethylenoxy chain with the use of longer PEG_400_ (DT1, Figure 1). However, alternative modifications include simple replacement of the acrylate function on the ethylenoxy fragment with an acrylamide moiety (DT2, Figure 1), which is dictated by the significantly higher hydrophilicity of acrylamides compared to acrylates. For this study acrylamide (AM), *N*-(2-hydroxyethyl)acrylamide (HEAM), or *N*,*N*-dimethylacrylamide (DMAM) were considered as main monomers to form hydrogels. The complete dissolution of diacetal crosslinkers was possible only with the least hydrophilic DMAM. This indicates that DMAM acts as a “cosolvent” for diacetals, influencing solubility in aqueous media. Based on this result, DMAM was selected as the main monomer for the fabrication of acid-degradable disc-shaped hydrogels. For both sets of trehalose acetalic monomers, MT1/DT1 and MT2/DT2, three comparable compositions of monomers were used, by keeping a constant diacetal concentration and varying DMAM and monoacetal content (Table S1). The solubility of trehalose monoacetals in the monomers feed was determined as ~40 wt%. However, this concentration causes a high content of hydrophobic aromatic rings that remain bound to the copolymers after hydrogel degradation, which leads to aqueous insolubility of degradation products. Finally, the maximum content of trehalose monoacetals in monomer compositions that still promote hydrogel degradation in aqueous soluble copolymers was 30 wt%. The total content of trehalose built into the hydrogels was examined by enzymatic method using Trehalose Assay Kit after cleavage from the polymer networks into solution via hydrolysis of the acetal groups in strong acidic conditions. Confirmation of the complete cleavage of trehalose under such conditions was shown by ^1^H NMR spectroscopy (Figure 2B,C,E and Figures S8 and S9) displaying sharp multiplets originating from the free trehalose, as well as broad signals from residual benzaldehyde parts of acetals attached to DMAM copolymers. The determined trehalose content was in the range of 1.7 to 11.4 and 2.0 to 16.4 wt% (Table 1) for hydrogels with MT1/DT1 and MT2/DT2 set, respectively. Considering monoacetals with the same w/w ratio, incorporation of higher amounts of trehalose was possible for amide trehalose monomer MT2 in comparison to polyethylenoxy analogue MT1 due to lower molecular weight of its aldehyde part and higher molar content of trehalose. Based on the integral values of aromatic signals of aldehyde and anomeric signals of trehalose in ^1^H NMR spectra of post-degradation solution (Figure S7) the content of trehalose originating from mono- and diacetal are distinguishable (Table 1, for calculations see Section S3.3 in Supporting Material). Firstly, an increase in the amount of monoacetals promotes the incorporation of greater amounts of diacetals. Moreover, higher amount of amide type diacetal DT2, than polyethylenoxy analogue DT1 in the corresponding hydrogels indicates higher crosslinking degree of the former materials.

**Figure 3:**
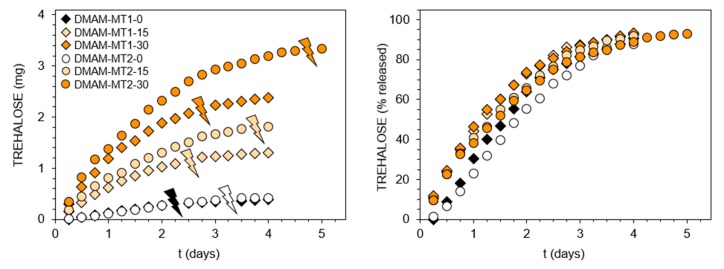
Trehalose release profiles from *N*,*N*-dimethylacrylamide (DMAM)-based hydrogels containing benzylidene acetals of trehalose at pH 5.0, 37 °C. Thunder signs show visual degradation of hydrogels into aqueous soluble products.

The enzymatic assay was also used to follow trehalose release in a mildly acidic environment of pH 5.0 at 37 °C, the obtained profiles are shown in Figure 3. Absolute amounts of releasing trehalose are in accordance with its content in the polymer networks (Figure 3, left side), with the highest being released from hydrogel DMAM-MT2-30. By recalculating this amount to the percentage of released trehalose (Figure 3, right side) additional conclusions were drawn, especially when analyzing the first 48 h. While no significant differences are observed in the percentage profiles corresponding to hydrogels differing in the amount of monoacetals, these hydrogels release trehalose significantly faster than hydrogels with trehalose bound only in the form of diacetal. This was expected, since monoacetals require cleavage of one acetal group to release trehalose, in contrary to diacetal’s two binding sites. Furthermore, trehalose was released slightly faster from hydrogels containing polyethylenoxy acetals MT1/DT1 than those with amide type acetals MT2/DT2. Most likely it is related to differences in both electron effects on the aromatic ring substituents as well as solvation effects resulting from overall structure hydrophilicity [25]. Accordingly, the significantly shorter time of hydrogel degradation into water-soluble products was observed for materials containing polyethylenoxy acetals MT1/DT1 when compared with the corresponding hydrogels with amide type acetals MT2/DT2 (Table 1, thunder sign in Figure 3). For example, hydrogel DMAM-MT1-30 degrades after 66 h, while hydrogel DMAM-MT2-30 requires 114 h. However, when trying to explain the differences in degradation time between these two sets of hydrogels it should be considered that hydrogels with polyethylenoxy acetals MT1/DT1 feature lower crosslinking degree as it was concluded earlier based on the calculated amount of trehalose originating from diacetals. Additionally, the increase in degradation time along with increasing amount of monoacetal for both sets of hydrogels seemed to result mainly from the crosslinking degree, since higher amount of monoacetals promotes incorporation of higher amount of diacetals. Another observation is that at the time of degradation of hydrogels into aqueous-soluble products (thunder sign, Figure 3) not all trehalose is released. This is due to the two-step hydrolysis of disubstituted trehalose derivatives. After hydrolysis of one acetal group visual disintegration occurs, however trehalose still remains linked with the dissolved polymer backbone as monoacetal and thus is not enzymatically detectable.

### 3.2. Hydrogels Capable of Trehalose Release under Slightly Alkaline Conditions

**Figure 4:**
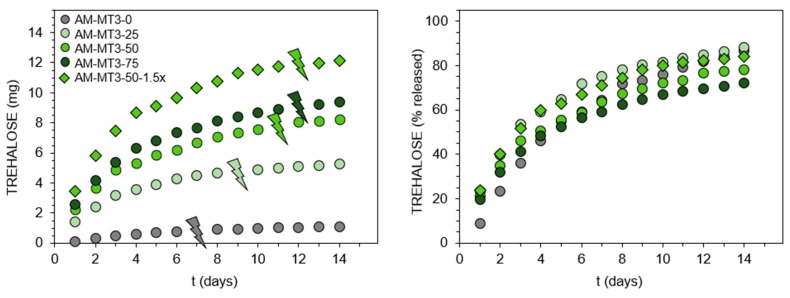
Trehalose release profiles from acrylamide (AM)-based hydrogels containing trehalose acrylates at pH 7.4, 37 °C. Thunder signs show visual degradation of hydrogels into aqueous soluble products.

Although trehalose release under mildly acidic conditions seems relatively easy by using trehalose acetals, release triggered by alkaline hydrolysis of esters at pH 7.4 is more challenging, owing to the ester resistance to cleavage under such conditions. Recently, we found this could be realized via the incorporation of the appropriate acrylamide-type comonomer [25]. It was shown that AM significantly accelerates hydrolysis of the ester moiety in the acrylate units. Therefore, in this study, the set of trehalose acrylates MT3/DT3 and AM was used to form hydrogels, which release trehalose at pH 7.4. Detailed feed compositions are given in Table S2. In contrast to benzylidene acetals, trehalose acrylates are freely soluble in the reaction medium, enabling a significant increase in the ratio of monosubstituted trehalose to main monomer. Additionally, simple acrylate functionalization results in higher molar content of trehalose in monomer molecules. These enabled to obtain hydrogels with significantly greater trehalose content in the range of 4.6 to 51.7 wt%. The complete release of trehalose into solution before its enzymatic determination was confirmed by ^1^H NMR spectroscopy (Figure 2D,E and Figure S10). Results of trehalose release study at pH 7.4, 37 °C are presented in Figure 4. Comparing the release profiles of hydrogels AM-MT3-25, AM-MT3-50, and AM-MT3-75 containing increasing content of trehalose, absolute amounts of trehalose released was in accordance with its content in the polymer networks (Figure 4, left side). However, when examining the release profiles expressed as a percentage of released trehalose (Figure 4, right side), the trend was reversed, the release rate decreased with increasing trehalose content. For example, after 7 days 75, 64, and 59% of trehalose was released from hydrogels AM-MT3-25, AM-MT3-50, and AM-MT3-75, respectively. This can be explained by the increased amount of trehalose monomers and thus reduced AM content, which plays a crucial role in the ester hydrolysis. 

Hydrogel AM-MT3-50-1.5x was prepared by taking AM and trehalose monoacrylate at 50/50 (w/w) ratio – the same as for hydrogel AM-MT3-50, but at 1.5 fold higher concentration of monomers, and thus the resulting hydrogel possessed higher polymer content. AM-MT3-50-1.5x contains also the same absolute amount of trehalose acrylate as AM-MT3-75, but features higher AM to trehalose monoacrylate ratio. By comparing releasing profiles of hydrogels AM-MT3-50 and AM-MT3-50-1.5x, higher concentrations of polymer in hydrogel promotes an increase in the release rate of trehalose. Whereas the comparison of hydrogel AM-MT3-75 and AM-MT3-50-1.5x shows that by keeping the amount of trehalose monoacrylate constant but increasing the amount of AM, it was possible to obtain hydrogel featuring faster trehalose release. Time necessary for hydrogels to degrade into aqueous soluble products increased with increasing content of monoacrylate (Table 1, thunder sign in Figure 3), which may result from decreasing AM content. However, it was also possible that hydrogels differ in crosslinking degree, but no signals in ^1^H NMR spectra were observed that would enable the appropriate calculation to distinguished weather trehalose originated from mono or diester.

## 4. Conclusions

For the first time, the synthesis of trehalose-rich polymers capable of releasing trehalose under physiologically relevant conditions was presented and evaluated on the example of hydrogels. This was accomplished by derivatization of trehalose into esters or benzylidene acetals bearing polymerizable functionalities, followed by copolymerization with the appropriately selected acrylamide-type monomers. Incorporation of trehalose into the polymer networks through benzylidene acetals resulted in hydrogels capable of trehalose release under mildly acidic conditions. By combining trehalose acrylates with AM, it was possible to develop materials that release trehalose at pH 7.4. The introduction of trehalose as pending moieties through trehalose derivatives with one polymerizable site, provides control over the amount of trehalose being released. Additionally, the use of derivatives possessing two polymerizable functionalities that act as crosslinkers, enables hydrogels to simultaneously release trehalose while undergoing degradation into aqueous soluble polymers. While alkali-labile hydrogels fabricated from trehalose acrylates and AM contained up to 51.7 wt% free trehalose, the maximum amount that can be incorporated into acid-labile hydrogels through trehalose benzylidene acetals was determined as 16.4 wt% since the limited solubility of benzylidene acetals as well as solubility of the degradation products. 

To sum up, in the current research we have devised the strategies of trehalose attachment to polymers to make it releasable in mildly acidic or slightly alkaline environment, which were presented on model hydrogel systems. This preliminary study provides the first basis for future research on polymeric materials intended for trehalose release upon hydrolysis under mild, physiologically relevant conditions, which could be attractive for both advanced biological studies on the mechanisms of autophagy regulation or practical application such as programmable release nanosystems for intravenous application.

## Figures and Tables

**Table 1:** Characteristics of hydrogels.

Hydrogel	DMAM or AM/MTx in The Feed (w/w) ^A^	Yield (%)	Calcd. Content of Trehalose in the Monomers Feed (wt%)	Detd. Content of Trehalose in the Hydrogel (wt%)	Detd. Content of Trehalose Originating from crosslinker ^B^ (wt%)	ESR	Degradation Time at 37 °C
Acid-labile hydrogels							
**DMAM-MT1-0**	100/0	88	2.4	1.7	1.7	20.6	54 h *
**DMAM-MT1-15**	85/15	83	7.6	6.1	2.1	19.7	60 h *
**DMAM-MT1-30**	70/30	81	12.9	11.4	2.5	19.1	66 h *
**DMAM-MT2-0**	100/0	88	2.6	2.0	2.0	19.6	78 h *
**DMAM-MT2-15**	85/15	85	11.5	8.5	2.3	17.7	90 h *
**DMAM-MT2-30**	70/30	80	20.3	16.4	3.0	17.0	114 h *
Alkali-labile hydrogels							
**AM-MT3-0**	100/0	96	5.7	4.6	ND	16.9	7 days **
**AM-MT3-25**	75/25	96	25.6	22.7	ND	17.0	9 days **
**AM-MT3-50**	50/50	91	45.6	39.9	ND	17.2	11 days **
**AM-MT3-75**	25/75	87	65.6	51.7	ND	22.8	12 days **
**AM-MT3-50-1.5x**	50/50	84	44.8	39.6	ND	13.8	12 days **

^A^ For detailed feed composition see ESI ^B^ calculated from ^1^H NMR spectra of degradation products (ESI) * at pH 5.0 ** at pH 7.4.

## Supplementary Materials

The following are available online at https://www.mdpi.com/2073-4360/11/12/2027/s1, synthetic procedures and ^1^H & ^13^C NMR spectra of trehalose monomers MT1/DT1, MT2/DT2, MT3/DT3, feed composition for the synthesis of hydrogels, detailed calculations of trehalose content in hydrogels, ^1^H NMR spectra of degradation products, Scheme S1: Synthetic pathway to trehalose monomers DT1 and MT1, Figure S1: ^1^H and ^13^C NMR spectra of 4,6-*O*-[*p*-(acryloyloxy(polyethylenoxy))benzylidene]-α,α’-D-trehalose (MT1), Figure S2: ^1^H and ^13^C NMR spectra of 4,6:4’,6’-di-*O*-[*p*-(acryloyloxy(polyethylenoxy))benzylidene]-α,α’-D-trehalose (DT1), Scheme S2: Synthetic pathway to trehalose monomers DT2 and MT2, Figure S3: ^1^H and ^13^C NMR spectra of 4,6-*O*-[*p*-(2-acrylamidoethoxy)benzylidene]-α,α’-D-trehalose (MT2), Figure S4: ^1^H and ^13^C NMR spectra of 4,6:4’,6’-di-*O*-[*p*-(2-acrylamidoethoxy)benzylidene]-α,α’-D-trehalose (DT2), Scheme S3: Synthetic pathway of trehalose monomers DT3 and MT3, Figure S5: ^1^H and ^13^C NMR spectra of 6-*O*-acryloyl-α,α’-D-trehalose (MT3), Figure S6: ^1^H and ^13^C NMR spectra of 6,6’-di-*O*-acryloyl-α,α’-D-trehalose (DT3), Table S1: Monomer feed compositions and hydrogels yields, Table S2: Monomer feed compositions and hydrogels yields, Figure S7: Signals in ^1^H NMR spectra of degradation products of acid-labile hydrogels used for estimation of trehalose content originating from mono- and diacetal, Table S3: Estimation of trehalose content originating from mono- and diacetal in acid-labile hydrogels, Figure S8: ^1^H NMR spectra of degradation products of acid-labile hydrogels containing trehalose monomers set MT1/DT1, Figure S9: ^1^H NMR spectra of degradation products of acid-labile hydrogels containing trehalose monomers set MT2/DT2, Figure S10: ^1^H NMR spectra of degradation products of alkali-labile hydrogels containing trehalose monomers set MT3/DT3.
