# Supplementary material for: Trehalose-Rich, Degradable Hydrogels Designed for Trehalose Release under Physiologically Relevant Conditions
Source: Polymers (Basel). 2019 Dec 6;11(12):2027. doi: 10.3390/polym11122027 (PMC6960900; doi:10.3390/polym11122027)
Supplement: Supplementary file 1 [file polymers-11-02027-s001.pdf]

Supporting Material

Trehalose-rich, degradable hydrogels designed for trehalose release under physiologically relevant conditions

Małgorzata Burek[1,2], Ilona Wandzik*[1,2]

[1]*Department of Organic Chemistry, Bioorganic Chemistry and Biotechnology, Faculty of Chemistry, Silesian University of Technology, B. Krzywoustego 4, 44 100 Gliwice, Poland*
[2] *Biotechnology Center, Silesian University of Technology, B. Krzywoustego 8, 44 100 Gliwice, Poland*

*Correspondence to: I. Wandzik, e-mail: ilona.wandzik@polsl.pl

EXPERIMENTAL

1. Materials and general methods S2
2. Synthesis and characterization of trehalose monomers S3
   2.1. Synthetic procedure for **MT1** and **DT1** S3
   2.2. Synthetic procedure for **MT2** and **DT2** S8
   2.3. Synthetic procedure for **MT3** and **DT3** S13
3. Synthesis and study on hydrogels..S19
   3.1. Feed composition for the synthesis of acid-labile hydrogels S19
   3.2. Feed composition for the synthesis of alkali-labile hydrogels S19
   3.3. Estimation of trehalose content originating from mono- and diacetal S19
4. References S24

1. Materials and general methods

Acrylamide (AM), acryloyl chloride, amberlyst-15, ammonia aqueous, ammonium persulphate (APS), 2,2'-Azobis[2-(2-imidazolin-2-yl)propane] dihydrochloride (V-044), Celite 501, hydrochloric acid, hydroquinone, *p*-hydroxybenzaldehyde, lithium tetrafluoroborate, methanesulfonyl chloride, phosphate buffered saline (PBS) tablets, polyethylene glycol (PEG_{400}), pyridine, silver(I) oxide (Ag_2O), sodium hydroxide, *N,N,N',N'*-tetramethylethylenediamine (TMEDA), *p*-toluenesulfonic acid, tosyl chloride, anhydrous trehalose, triethylamine (TEA), and trimethyl orthoformate, trimethylsilyl chloride were purchased from Sigma Aldrich, Acros Organics, TCI or Fluka and used directly without any purification. *N,N*-dimethylacrylamide (Sigma Aldrich) were purified by passing through a column filled with basic aluminum oxide to remove inhibitor. Anhydrous solvents were purchased from Acros Organics and stored over molecular sieves under inert atmosphere. All other solvents and inorganic salts were purchased from Avantor Performance Materials Poland S.A. Trehalose Assay Kit was purchased from Megazyme International Ireland.

p-(2-Hydroxyethoxy)benzaldehyde was synthesized according to the published procedure[1].

Purifications by flash column chromatography were performed on silica gel 60 (40-63 µm, Merck) using automated system (Isolera, Biotage). Freeze-drying was carried out under 0.035 mbar at -50 °C (ALPHA 1-2 LDplus, CHRIST). Mass spectra were recorded using Electrospray Ionisation Mass Spectrometry (QTRAP 4000, AB Sciex). NMR spectra were recorded in deuterated solvents (Deutero GmbH) with internal standards using NMR spectrometer operating at 400 or 600 MHz (Varian).

2. Synthesis and characterization of trehalose monomers

2.1. Synthetic procedure for MT1 and DT1

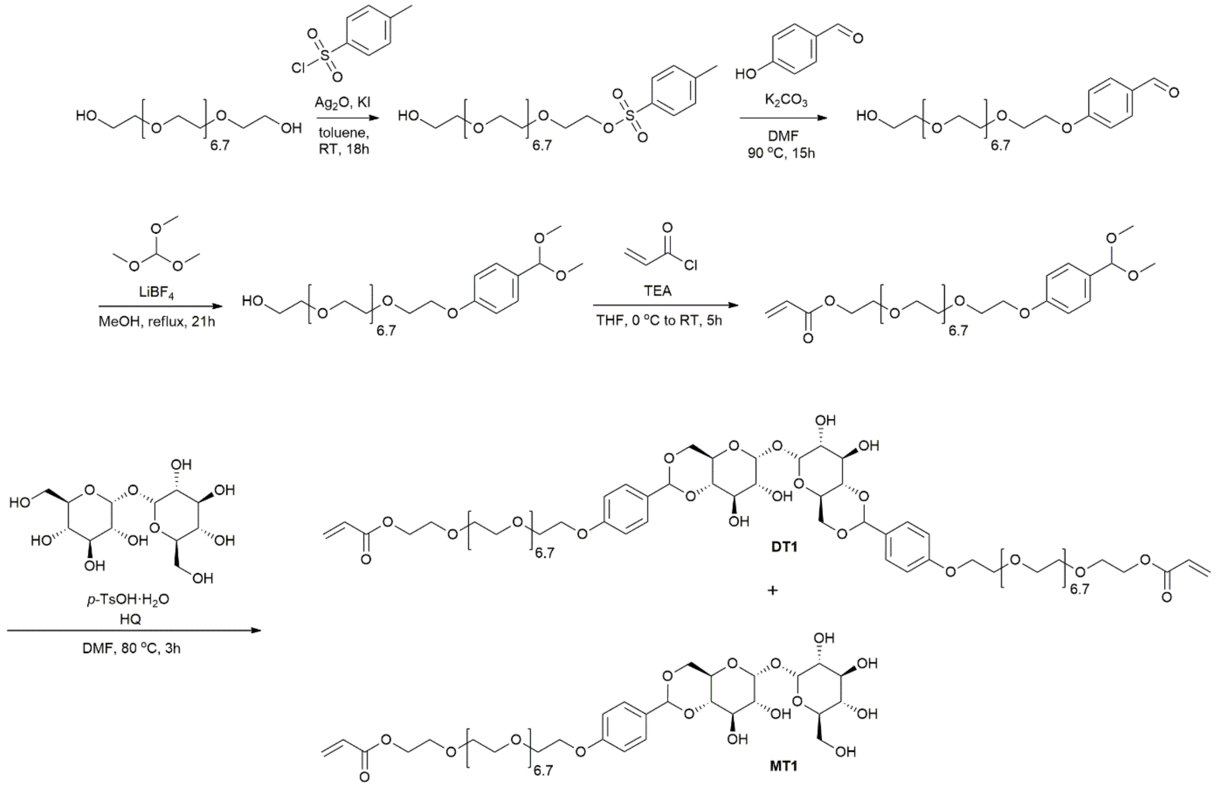

Scheme S1 Synthetic pathway to trehalose monomers DT1 and MT1.

p-(Hydroxy(polyethylenoxy))benzaldehyde

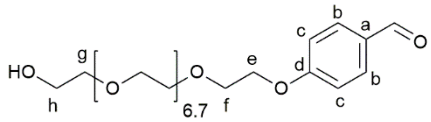

p-(2-Hydroxy(polyethylenoxy))benzaldehyde was synthesized in two step reaction starting from PEG_{400}, based on the slightly modified literature procedures[2,3].

Briefly, PEG_{400} (10.3 mmol) was dried by repeated evaporation with toluene under vacuum and then dissolved in 125 mL of anhydrous toluene. KI (2.1 mmol) and Ag_2O (15.4 mmol) were added and magnetic stirring was set to 1000 rpm before tosyl chloride (10.8 mmol) was quickly introduced in one portion. The reaction was left overnight and resulting suspension was then filtrated through the pad of Celite 501. Toluene was evaporated under vacuum to afford tosylated PEG_{400} as a viscous, colorless oil, which was subjected to the second step without further purification. After dissolving in 25 mL of anhydrous DMF, *p*-hydroxybenzaldehyde (15.5 mmol) and K_2CO_3 (20.6 mmol) were added and the reaction was left overnight under stirring at 90 °C. Afterwards, DMF was evaporated under vacuum, 50 mL of 5% Na_2CO_3 aqueous solution was added to the residue and the mixture was extracted with DCM (2 x 50 mL). The combined organic layers were washed with 5% Na_2CO_3 aqueous solution (2 x 50 mL) and brine (50 mL), dried over $MgSO_4$ and concentrated under vacuum to afford product as a yellow viscous oil (4.45 g). As calculated from ^1H NMR spectrum, the product was found to contain 76.3 mol%

and 23.7 mol% of mono- and disubstituted PEG_{400} derivatives, respectively. The product was taken forward without further purification. Monosubstituted PEG_{400} derivative was separated from disubstituted PEG_{400} derivative after next step (acetalization with trimethylorthoformate) by silica gel column chromatography.

^{1}H NMR* (DMSO-d_6, 600 MHz,) δ [ppm]: 3.39-3.42 (m, 2H, H_h); 3.44-3.63 (m, ~29 H, H_g, PEG backbone); 3.74-3.80 (m, 2H, H_f); 4.19-4.24 (m, 2H, H_e); 4.53-4.60 (m, 1H, –OH); 7.10-7.17 (m, 2H, H_c); 7.83-7.89 (m, 2H, H_b); 9.86 (br s, 1H, –CHO). **^{13}C NMR** (DMSO-d_6, 150 MHz) δ [ppm]: 60.20 (C_h); 67.68, 68.69, (C_e, C_f); 69.66-69.96 (PEG backbone); 72.33 (C_g); 114.95 (C_c);; 129.64 (C_a); 131.78 (C_b) 163.48 (C_d). 191.29 (–CHO).

*with respect to monosubstituted PEG_{400} derivative. Molar ratio of mono- to disubstituted PEG_{400} derivative: (1 : 0.31)

p-(Hydroxy(polyethylenoxy))benzaldehyde dimethyl acetal

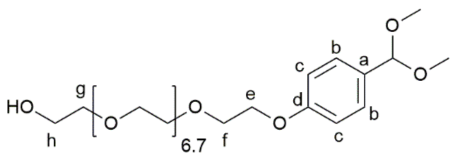

p-(Hydroxy(polyethylenoxy))benzaldehyde dimethyl acetal was synthetized based on the reported procedure.[4] To a solution of *p*-(hydroxy(polyethylenoxy))benzaldehyde (4.40 g) and lithium tetrafluoroborate (0.9 mmol) in methanol (20 mL), trimethyl orthoformate (17.6 mmol) was introduced, and the solution was stirred under reflux for 21h. After cooling, pyridine (0.19 mmol) and saturated aqueous solution of $NaHCO_3$ (50 mL) was added, and the mixture was extracted twice with DCM (70 mL). Organic layer was then washed with saturated aqueous solution of $NaHCO_3$ (40 mL), dried over $MgSO_4$ and concentrated under vacuum. The crude product was purified by silica gel flash chromatography ($CHCl_3$: TEA 99:1) to afford product as a yellowish oil (3.68 g, 65%*).

^{1}H NMR (DMSO-d_6, 600 MHz) δ [ppm]: 3.21 (s, 6H, $–CH_3$); 3.39-3.43 (m, 2H, H_h); 3.45-3.60 (m, ~29 H, H_g, PEG backbone); 3.72-3.75 (m, 2H, H_f); 4.06-4.10 (m, 2H, H_e); 4.54-4.58 (m, 1H, –OH); 5.31 (s, 1H, –OCHO–); 6.91-6.95 (m, 2H, H_c); 7.26-7.31 (m, 2H, H_b). **^{13}C NMR** (DMSO-d_6, 150 MHz) δ [ppm]: 52.31 ($–CH_3$); 60.20 (C_h); 67.09, 68.90, (C_e, C_f); 69.70-69.98 (PEG backbone); 72.33 (C_g); 102.47 (–OCHO–); 113.94 (C_c); 127.77 (C_b); 130.35 (C_a); 158.40 (C_d).*after 3 steps

p-(Acryloyloxy(polyethylenoxy))benzaldehyde dimethyl acetal

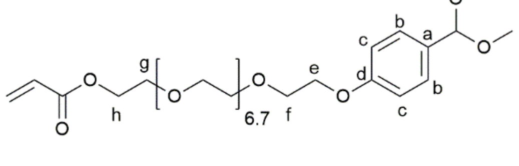

A solution of p-(hydroxy(polyethylenoxy))benzaldehyde dimethyl acetal (6.5 mmol) and TEA (14.6 mmol) in THF (80 mL) was cooled on ice water bath, and then a solution of acryloyl chloride (9.8 mmol) in THF (5 mL) was added dropwise. The mixture was left to warm up to room temperature and the reaction was finished after 3 h. Precipitated triethylamine hydrochloride was separated on a pad of Celite 501, and filtrate was concentrated under vacuum. The crude product was purified by silica gel flash

chromatography ($CHCl_3$: TEA 99:1) to afford *p*-(acryloyloxy(polyethylenoxy))benzaldehyde dimethyl acetal as a yellowish syrup (3.61 g, 92%).

^{1}H NMR (DMSO-d$_6$, 600 MHz) δ [ppm]: 3.21 (s, 6H, $-CH_3$); 3.48-3.60 (m, ~27 H, PEG backbone); 3.62-3.66 (m, 2H, H_g); 3.72-3.75 (m, 2H, H_f); 4.06-4.10 (m, 2H, H_e); 4.19-4.23 (m, 2H, H_h); 5.31 (s, 1H, –OCHO–); 5.93-5.97 (m, 1H, –CH=C**H**$_2$ *cis*); 6.16-6.22 (m, 1H, –C**H**=CH$_2$); 6.30-6.36 (m, 1H, –CH=C**H**$_2$ *trans*); 6.90-6.95 (m, 2H, H_c); 7.26-7.30 (m, 2H, H_b). **^{13}C NMR** (DMSO-d$_6$, 150 MHz) δ [ppm]: 52.31 ($-CH_3$); 63.45 (C_g); 67.09, 68.91, (C_e, C_f); 68.21 (C_h); 69.65-69.95 (PEG backbone); 102.46 (–OCHO–); 113.93 (C_c); 127.77 (C_b); 128.21 (–**C**H=CH$_2$); 130.35 (C_a); 131.66 (–CH=**C**H$_2$); 158.41 (C_d); 165.47 (–OC(O)–).

4,6-*O*-[*p*-(acryloyloxy(polyethylenoxy))benzylidene]-α,α'-D-trehalose (MT1) and 4,6:4',6'-di-*O*-[*p*-(acryloyloxy(polyethylenoxy))benzylidene]-α,α'-D-trehalose (DT1)

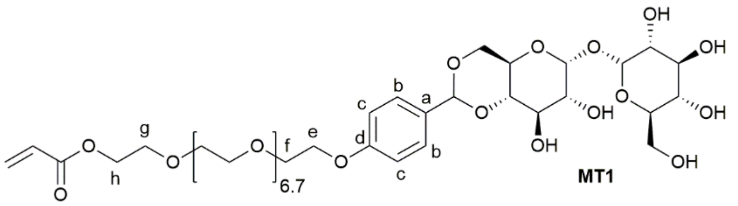

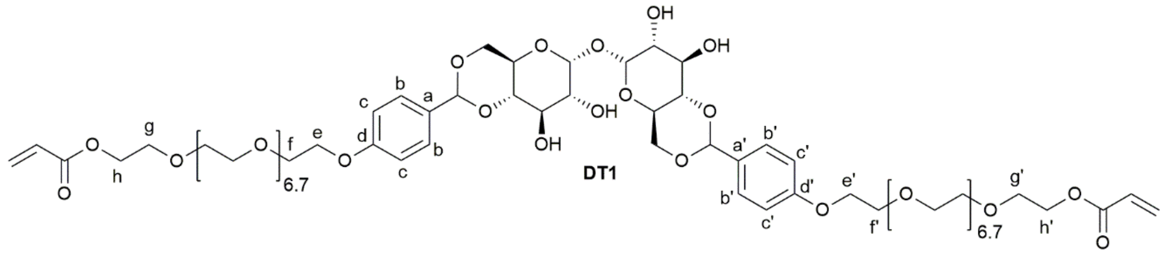

To a suspension of anhydrous trehalose (2.4 mmol) in anhydrous DMF (5 mL), p-(acryloyloxy(polyethylenoxy))benzaldehyde dimethyl acetal (5.5 mmol), hydroquinone (0.05 mmol) and a catalytic amount of *p*-toluenesulfonic acid were added. The solution was stirred at 80 °C for 3 h and then reaction was quenched with TEA. DMF was evaporated under reduced pressure and the sticky residue was treated with diethyl ether (3 x 50 mL). **DT1** and **MT1** were separated by silica gel flash chromatography ($CHCl_3$: MeOH : TEA 97:2 :1 → 88:11:1) as yellowish oils (1.54 g, 45% and 0.28 g, 13%, respectively).

4,6-O-[p-(acryloyloxy(polyethylenoxy))benzylidene]-α,α'-D-trehalose (**MT1**)

^{1}H NMR (DMSO-d$_6$, 600 MHz) δ [ppm]: 3.15 (dd, 1H, J = 9.6, 5.2 Hz, H-4'); 3.26 (ddd, 1H, J = 9.6, 5.8, 3.7 Hz, H-2'); 3.34 (~t, 1H, J = 9.6 Hz, H-4); 3.39 (ddd, 1H, J = 9.8, 6.4, 3.8 Hz, H-2); 3.45-3.67 (m, ~33 H, H-3', H-6'a, H-6'b, H-6a, PEG backbone, H_g); 3.70 (ddd, 1H, J = 10.9, 4.8, 2.3 Hz, H-5'); 3.72-3.76 (m, 3H, H-3, H_f); 3.98 (~td, 1H, J = 9.9, 5.0 Hz, H-5); 4.04-4.10 (m, 3H, H-6b, H_e); 4.19-4.24 (m, 2H, H_h); 4.36 (~t, 1H, J = 6.0 Hz, C-6'-OH'); 4.75-4.79 (m, 2H, 2x –OH); 4.87 (d, 1H, J = 3.7 Hz, H-1'); 4.90-4.95 (m, 3H, H-1, 2x –OH); 5.16 (d, 1H, J = 5.1 Hz, –OH); 5.49 (s, 1H, –OCHO–); 5.92-5.98 (m, 1H, –CH=C**H**$_2$ *cis*); 6.15-6.23 (m, 1H, –C**H**=CH$_2$); 6.30-6.36 (m, 1H, –CH=C**H**$_2$, *trans*); 6.88-6.95 (m, 2H, H_c); 7.31-7.38 (m, 2H, H_b). **^{13}C NMR** (DMSO-d$_6$, 150 MHz) δ [ppm]: 63.44 (C_g); 67.14, 68.89 (C_e, C_f); 68.21 (C_h); 69.60-69.94 (PEG backbone); 60.69, 62.40, 68.21, 69.52, 70.00, 71.47, 72.13, 72.62, 72.71, 81.47 (C-2, C-2', C-3, C-3', C-4, C-4', C-5, C-5', C-6, C-6'); 93.81, 94.21 (C-1, C-1'); 100.72 (–OCHO–); 113.81 (C_c); 127.67 (C_b); 128.20 (–**C**H=CH$_2$); 130.31 (C_a); 131.63 (–CH=**C**H$_2$); 158.69 (C_d); 165.45 (–C(O)O–).

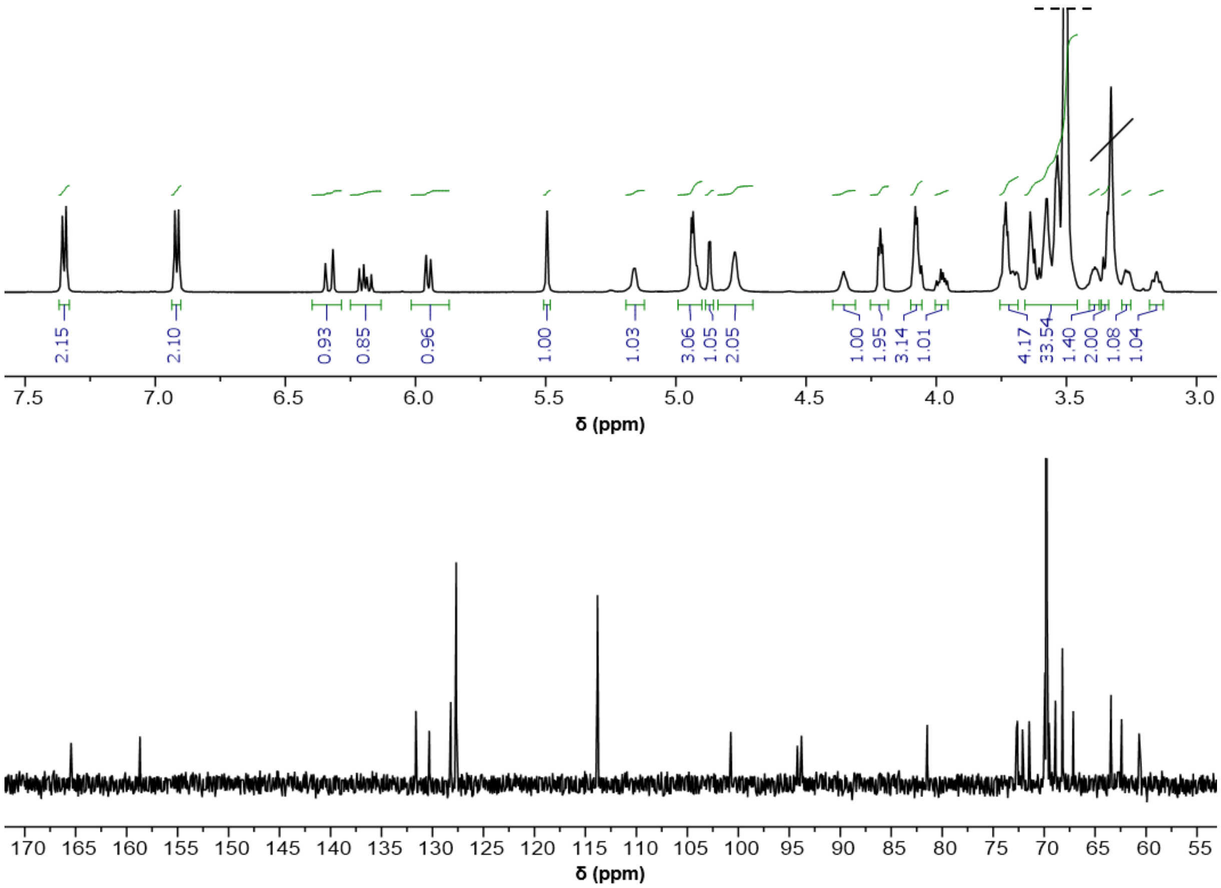

Fig. S1 ^{1}H and ^{13}C NMR spectra of 4,6-*O*-[*p*-(acryloyloxy(polyethylenoxy))benzylidene]-α,α'-D-trehalose **(MT1)**

4,6:4',6'-di-O-[p-(acryloyloxy(polyethylenoxy))benzylidene]-α,α'-D-trehalose **(DT1)**

^{1}**H NMR** (DMSO-d$_6$, 600 MHz) δ [ppm]: 3.36 (~t, 2H, J = 9.4 Hz, H-4, H-4'); 3.42 (ddd, 2H, J = 9.6, 6.1, 3.8 Hz, H-2, H-2'); 3.47-3.60 (m, ~54 H, PEG backbone); 3.60-3.66 (m, 6H, H-6a, H-6'a, H_g, $H_{g'}$); 3.71-3.79 (m, 6H, H-3, H-3', H_f, $H_{f'}$); 4.00-4.10 (m, 8H, H-5, H-5', H-6b, H-6'b, H_e, $H_{e'}$); 4.19-4.23 (m, 4H, H_h, $H_{h'}$); 4.93 (d, 2H, J = 3.8 Hz, H-1, H-1'); 5.18 (d, 2H, J = 5.1 Hz, C-3-OH, C-3'-OH'); 5.25 (d, 2H, J = 6.0 Hz, C-2-OH, C-2'-OH'); 5.50 (s, 2H, –OCHO–, –OC'H'O–); 5.91-5.98 (m, 2H, –CH=C**H**$_2$, –C'H'=C'**H'**$_2$ *cis*); 6.14-6.23 (m, 2H, –C**H**=CH$_2$, –C'**H'**=C'H'$_2$); 6.29-6.36 (m, 2H, –CH=C**H'**$_2$, –C'H'=C'**H'**$_2$ *trans*); 6.87-6.95 (m, 4H, H_c, $H_{c'}$); 7.31-7.39 (m, 4H, H_b, $H_{b'}$). ^{13}**C NMR** (DMSO-d$_6$, 150 MHz) δ [ppm]: 63.44 (C_g, $C_{g'}$); 67.13, 68.89 (C_e, C_e', C_f, C_f'); 68.20 (C_h, $C_{h'}$); 69.65-69.95 (PEG backbone); 62.57, 68.20, 69.42, 72.00, 81.42 (C-2, C-2', C-3, C-3', C-4, C-4', C-5, C-5', C-6, C-6'); 94.92 (C-1, C-1'); 100.75 (–OCHO–,–OC'H'O–); 113.81 (C_c, C_c'); 127.67 (C_b, C_b'); 128.20 (–**C**H=CH$_2$, –**C'**H'=C'H'$_2$); 130.29 (C_a, C_a'); 131.63 (–CH=**C**H$_2$, –C'H'=**C'**H'$_2$); 158.69 (C_d, C_d'); 165.45 (–C(O)O–, –C'(O)O–).

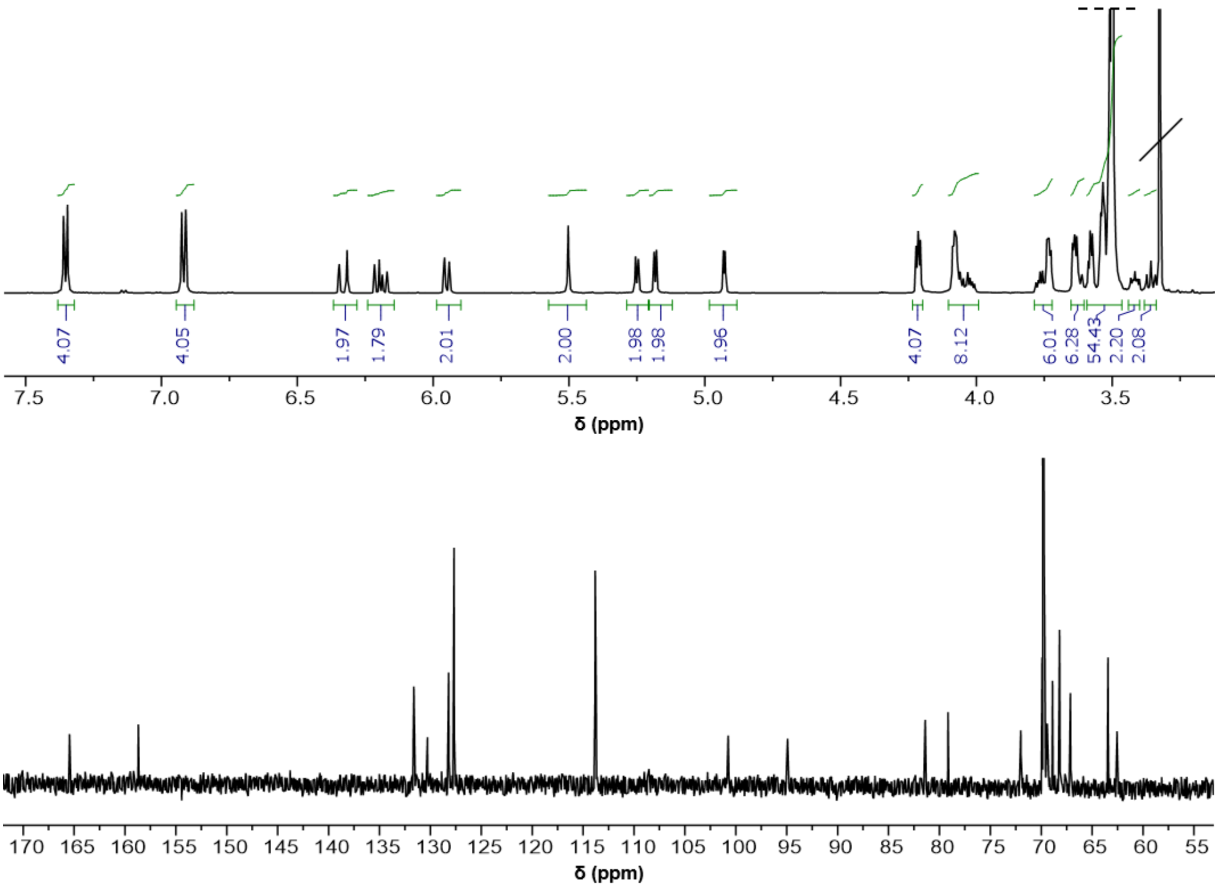

Fig. S2 ^{1}H and ^{13}C NMR spectra of 4,6:4′,6′-di-*O*-[*p*-(acryloyloxy(polyethylenoxy))benzylidene]-α,α′-D-trehalose (DT1)

2.2. Synthetic procedure for MT2 and DT2

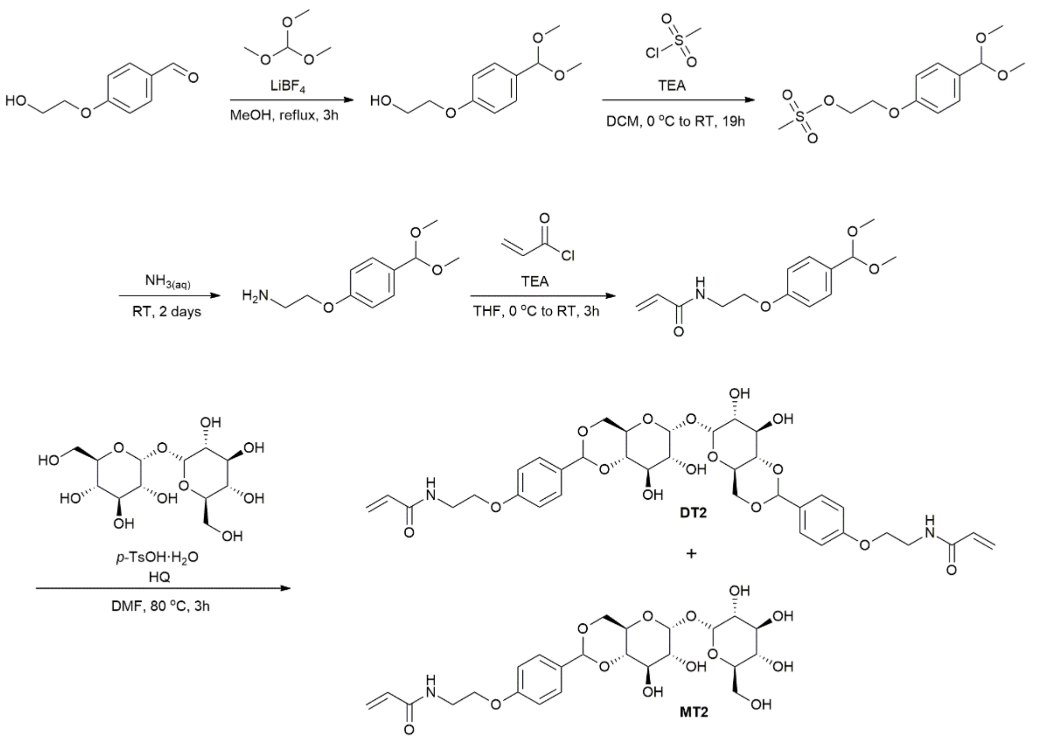

Scheme S2 Synthetic pathway to trehalose monomers **DT2** and **MT2**.

p-(2-Hydroxyethoxy)benzaldehyde dimethyl acetal

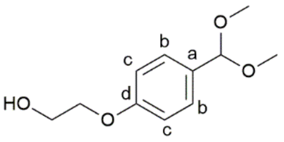

p-(2-Hydroxyethoxy)benzaldehyde dimethyl acetal was synthetized based on the reported procedure.[4] To a solution of *p*-(2-hydroxyethoxy)benzaldehyde (78.0 mmol) and lithium tetrafluoroborate (4 mmol) in methanol (40 mL), trimethyl orthoformate (117.0 mmol) was added, and the solution was stirred under reflux for 3h. After cooling, saturated aqueous solution of $NaHCO_3$ (250 mL) was added, and the mixture was extracted twice with ethyl acetate (360 mL, 120 mL). The combined organic layers were washed with saturated aqueous solution of $NaHCO_3$ (120 mL), dried over $MgSO_4$ and concentrated under vacuum to afford product as an orange syrup (15.92 g, 96%).

^{1}H NMR ($CDCl_3$, 600 MHz) δ [ppm]: 2.28 (t, 1H, J = 6.2 Hz, –OH); 3.31 (s, 6H, –OCH_3); 3.93-3.97 (m, 2H, –CH_2OH); 4.06-4.10 (m, 2H, –OCH_2–); 5.35 (s, 1H, –OCHO–); 6.89-6.93 (m, 2H, H_c); 7.35-7.39 (m, 2H, H_b). **^{13}C NMR** ($CDCl_3$, 150 MHz) δ [ppm]: 52.74 (–OCH_3); 61.59 (-CH_2OH); 69.36 (–OCH_2–); 103.12 (–OCHO–); 114.33 (C_c); 128.14 (C_b); 131.04 (C_a); 158.86 (C_d). **LR ESI-MS:** m/z calcd for $C_{11}H_{16}O_4Na$ $[M+Na]^+$ 235.1, found 235.2

p-(2-(Methanesulfonyloxy)ethoxy)benzaldehyde dimethyl acetal

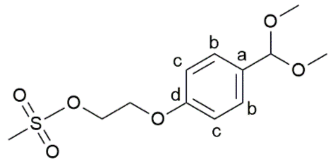

A solution of *p*-(2-hydroxyethoxy)benzaldehyde dimethyl acetal (30.0 mmol) and TEA (51.0 mmol) in anhydrous DCM (100 mL) was cooled on ice water bath, and then methanesulfonyl chloride (45.0 mmol) was added dropwise, while stirring. The mixture was left to warm up to room temperature and left overnight. Afterwards, the suspension was diluted with DCM (100 mL) and extracted with an aqueous solution of $NaHCO_3$ (100 mL). The organic layer was dried over $MgSO_4$ and concentrated under vacuum to afford product as an yellow syrup (7.99 g, 92%).

^{1}H NMR ($CDCl_3$, 600 MHz) δ [ppm]: 3.09 (s, 3H, $–SO_2CH_3$); 3.31 (s, 6H, $–OCH_3$); 4.23-4.27 (m, $–OCH_2–$); 4.55-4.59 (m, 2H, 2H, $–CH_2SO_2–$); 5.35 (s, 1H, –OCHO–); 6.87-6.92 (m, 2H, H_c); 7.36-7.40 (m, 2H, H_b). **^{13}C NMR** ($CDCl_3$, 150 MHz) δ [ppm]: 37.92 ($–SO_2CH_3$); 52.76 ($–OCH_3$); 65.96 ($–CH_2SO_2–$); 68.11 ($–OCH_2–$); 102.99 (–OCHO–); 114.32 (C_c); 128.26 (C_b); 131.60 (C_a); 158.17 (C_d). **LR ESI-MS:** m/z calcd for $C_{12}H_{18}O_6SNa$ $[M+Na]^+$ 313.1, found 313.2

p-(2-Aminoethoxy)benzaldehyde dimethyl acetal

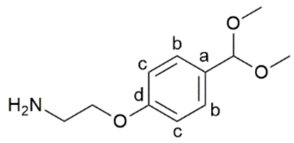

Aqueous ammonia (285 mL) was added to *p*-(2-(methanesulfonyloxy)ethoxy)benzaldehyde dimethyl acetal (25.0 mmol) and the solution was stirred vigorously for 2 days. The mixture was then extracted with DCM (250 mL), and afterwards the organic layer was washed twice with an aqueous solution of $NaHCO_3$ (250 mL), dried over $MgSO_4$ and concentrated under vacuum to afford product as an yellow syrup (5.11 g, 96%).

^{1}H NMR ($CDCl_3$, 600 MHz) δ [ppm]: 1.65 (br s, 2H, $–NH_2$); 3.08 (~t, 2H, J = 5.2, 5.2 Hz, $–CH_2NH_2$); 3.30 (s, 6H, $–OCH_3$); 3.99 (~t, 2H, J = 5.2, 5.2 Hz, $–OCH_2–$); 5.34 (s, 1H, –OCHO–); 6.86-6.92 (m, 2H, H_c); 7.32-7.38 (m, 2H, H_b). **^{13}C NMR** ($CDCl_3$, 150 MHz) δ [ppm]: 41.66 ($–CH_2NH_2$); 52.72 ($–OCH_3$); 70.20 ($–OCH_2–$); 103.16 (–OCHO–); 114.27 (C_c); 128.08 (C_b); 130.71 (C_a); 159.12 (C_d). **LR ESI-MS:** m/z calcd for $C_{11}H_{18}O_3N$ $[M+H]^+$ 212.1, found 212.2

p-(2-acrylamidoethoxy)benzaldehyde dimethyl acetal

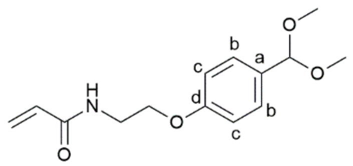

A solution of *p*-(2-aminoethoxy)benzaldehyde dimethyl acetal (24.0 mmol) and TEA (54.0 mmol) in THF (120 mL) was cooled on ice water bath, and then a solution of acryloyl chloride (36.0 mmol) in THF (10 mL) was added dropwise. The mixture was left to warm up to room temperature and the reaction was finished after 3 h. Precipitated triethylamine hydrochloride was separated on a pad of Celite 501, and

filtrate was concentrated under vacuum. The crude product was purified by silica gel flash chromatography ($CHCl_3$: TEA 99:1) to afford *p*-(2-acrylamidoethoxy)benzaldehyde dimethyl acetal as a yellowish syrup (3.65 g, 57%).

^{1}**H NMR** ($CDCl_3$, 600 MHz) δ [ppm]: 3.30 (s, 6H, –OCH_3); 3.74 (~q, 2H, J = 5.6 Hz, –CH_2NH–); 4.07 (~t, 2H, J = 5.1, 1.2 Hz, –OCH_2–); 5.33 (s, 1H, –OCHO–); 5.64 (dd, 1H, J = 10.2, 1.5 Hz, –CH=C**H**$_2$, *cis*); 6.12 (1H, J = 17.0, 10.2 Hz, –C**H**=CH_2); 6.13-6.25 (br s, 1H, –NHC(O)–); 6.29 (dd, 1H, J = 17.1, 2.2 Hz, –CH=C**H**$_2$, *trans*); 6.84-6.91 (m, 2H, H_c); 7.32-7.38 (m, 2H, H_b). ^{13}**C NMR** ($CDCl_3$, 150 MHz) δ [ppm]: 39.13 (–CH_2NH–); 52.73 (–OCH_3); 66.83 (–OCH_2–); 103.07 (–OCHO–); 114.19 (C_c); 126.88 (–CH=**C**H_2); 128.17 (C_b); 130.75 (–**C**H=CH_2); 131.11 (C_a); 158.64 (C_d); 165.78 (–OC(O)–). **LR ESI-MS:** m/z calcd for $C_{11}H_{18}O_3Na$ $[M+Na]^+$ 288.1, found 288.3

4,6:4′,6′-di-*O*-[*p*-(2-acrylamidoethoxy)benzylidene]-α,α′-D-trehalose (DT2) and 4,6-*O*-[*p*-(2-acrylamidoethoxy)benzylidene]-α,α′-D-trehalose (MT2)

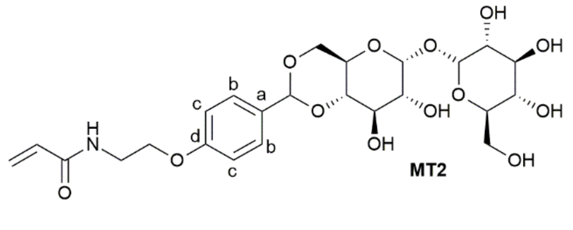

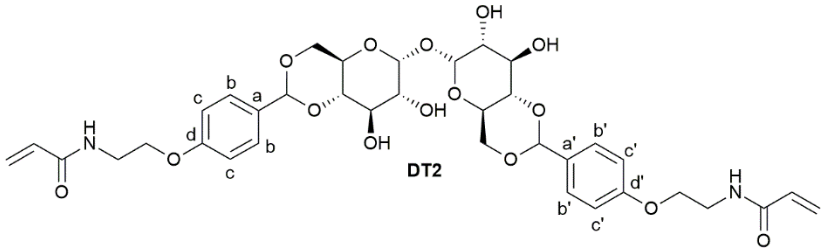

To a suspension of anhydrous trehalose (5.65 mmol) in anhydrous DMF (10 mL), *p*-(2-acrylamidoethoxy)benzaldehyde dimethyl acetal (13.0 mmol), hydroquinone (0.1 mmol) and a catalytic amount of *p*-toluenesulfonic acid were added. The solution was stirred for 3 h at 80 °C and then reaction was quenched with TEA. The solvent was evaporated under reduced pressure and the sticky residue was treated with diethyl ether (3 x 50 mL). **DT2** and **MT2** were separated by silica gel flash chromatography ($CHCl_3$: MeOH : TEA 97:2 :1 → 85:14:1) as white solids (1.85 g, 44% and 0.36 g, 12%, respectively).

4,6-O-[p-(2-acrylamidoethoxy)benzylidene]-α,α′-D-trehalose (**MT2**)

^{1}**H NMR** (DMSO-d_6, 600 MHz) δ [ppm]: 3.15 (~t, 1H, J = 9.4, Hz, H-4′); 3.24-3.29 (m, 1H, H-2′); 3.34-3.53 (m, 5H, H-4, H-2, H-6′a, –CH_2NH–); 3.53-3.60 (m, 2H, H-3′, H-6′b); 3.63 (~t, 1H, J = 10.2 Hz, H-6a); 3.70 (ddd, 1H, J = 10.0, 4.8, 2.3 Hz, H-5′); 3.74 (~t, 1H, J = 9.9 Hz, H-3); 3.98 (~td, 1H, J = 10.0, 5.0 Hz, H-5); 4.03 (~t, 2H, J = 5.63 Hz, –OCH_2–); 4.07 (dd, 1H, J = 10.0, 4.9 Hz, H-6b); 4.36 (br s, 1H, -OH); 4.60-5.10 (m, 4H, 4x –OH) 4.87 (d, 1H, J = 3.6 Hz, H-1′); 4.94 (d, 1H, J = 3.7 Hz, H-1); 5.20 (br s, 1H, J = 5.1 Hz, –OH); 5.50 (s, 1H, –OCHO–); 5.59 (dd, 1H, J = 10.2, 2.2 Hz, –CH=C**H**$_2$ *cis*); 6.10 (dd, 1H, J = 17.1, 2.2 Hz, –CH=C**H**$_2$, *trans*); 6.26 (dd, 1H, J = 17.1, 10.2 Hz, –C**H**=CH_2); 6.93 (d, 2H, J = 8.8 Hz, H_c, H_c′); 7.36 (d, 2H, J = 8.7 Hz, H_b, H_b′); 8.35 (t, 2H, J = 5.7 Hz, –NHC(O)–); ^{13}**C NMR** (DMSO-d_6, 150 MHz) δ [ppm]: 38.27 (–CH_2NH–); 66.26 (–OCH_2–, –OC′H′$_2$–); 60.70, 62.41, 68.22, 69.51, 70.01, 71.47, 72.15, 72.64, 72.71, 81.46 (C-2, C-2′, C-3, C-3′, C-4, C-4′, C-5, C-5′, C-6, C-6′); 93.84, 94.24 (C-1, C-1′); 100.70 (–OCHO–); 113.88 (C_c); 125.30

(–**C**H=CH$_2$); 127.71 (C$_b$); 130.46 (C$_a$) 131.57 (–CH=**C**H$_2$) 158.58 (C$_d$) 164.88 (–C(O)–). **LR ESI-MS:** m/z calcd for $C_{24}H_{33}O_{15}N_2$ [M+H]$^+$ 544.2, found 544.2

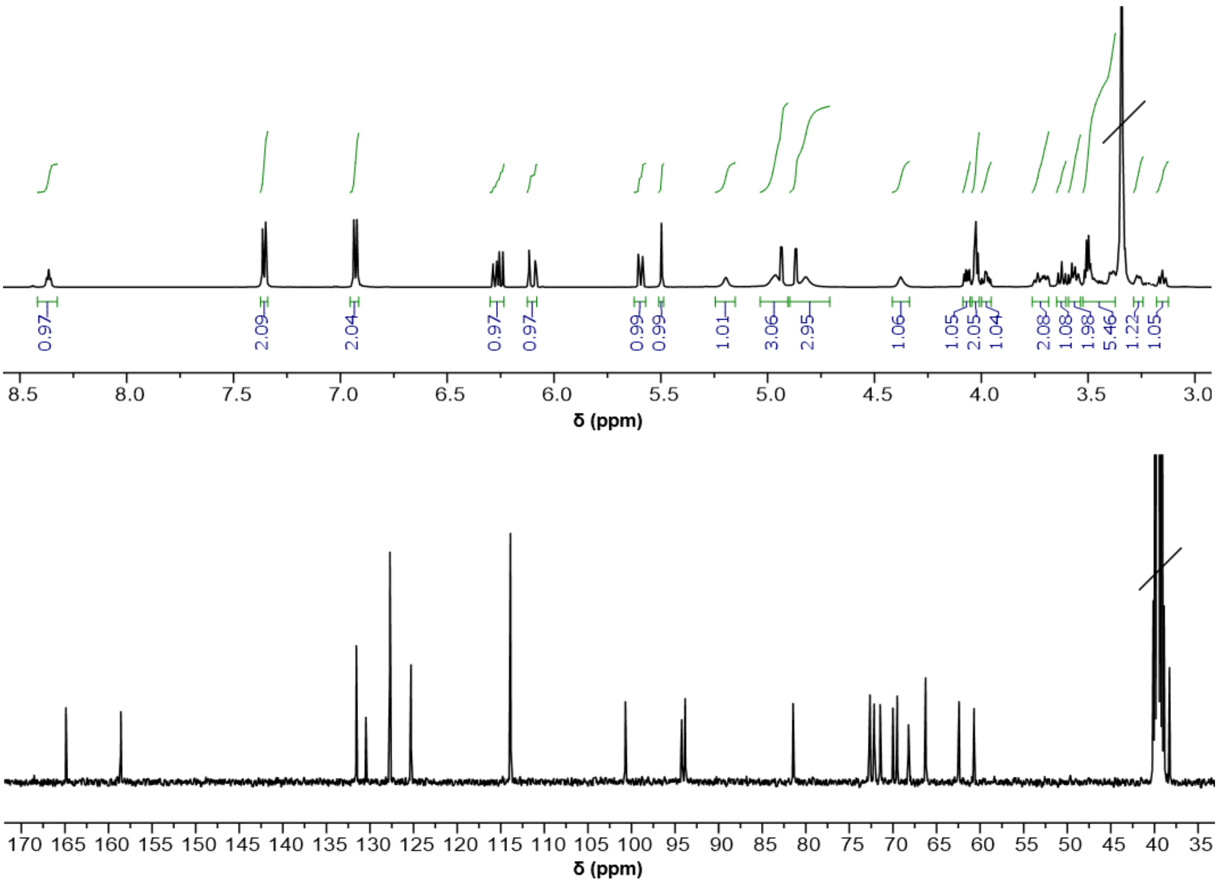

Fig. S3 ^{1}H and ^{13}C NMR spectra of 4,6-*O*-[*p*-(2-acrylamidoethoxy)benzylidene]-α,α'-D-trehalose **(MT2)**

4,6:4',6'-di-O-[p-(2-acrylamidoethoxy)benzylidene]-α,α'-D-trehalose **(DT2)**

^{1}**H NMR** (DMSO-d$_6$, 600 MHz) δ [ppm]: 3.36 (~t, 2H, J = 9.5 Hz, H-4, H-4');); 3.42 (ddd, 2H, J = 9.6, 6.0, 3.7 Hz, H-2, H-2'); 3.50 (~q, 2H, J = 5.6 Hz, –CH$_2$NH–, –C'H'$_2$NH'–); 3.63 (~t, 2H, J = 9.9 Hz, H-6a, H-6'a); 3.76 (~td, 2H, J = 9.3, 5.1 Hz, H-3, H-3'); 4.00-4.10 (m, 8H, H-5, H-5', H-6b, H-6'b, –OCH$_2$–, –OC'H'$_2$–); 4.93 (d, 2H, J = 3.8 Hz, H-1, H-1'); 5.19 (d, 2H, J = 5.1 Hz, C-3-OH, C-3'-OH'); 5.26 (d, 2H, J = 6.0 Hz, C-2-OH, C-2'-OH'); 5.51 (s, 2H, –OCHO–, –OCH'O–); 5.59 (dd, 2H, J = 10.2, 2.1 Hz, –CH=C**H**$_2$, –C'H'=C'**H'**$_2$ *cis*); 6.10 (dd, 2H, J = 17.1, 2.1 Hz, –CH=C**H**$_2$, –C'H'=C'**H'**$_2$ *trans*); 6.26 (dd, 2H, J = 17.1, 10.2 Hz, –C**H**=CH$_2$, –C'**H'**=C'H'$_2$); 6.93 (d, 4H, J = 8.7 Hz, H$_c$, H$_c$'); 7.36 (d, 4H, J = 8.7 Hz, H$_b$, H$_b$'); 8.36 (t, 2H, J = 5.7 Hz, –NHC(O)–, –NH'C'(O)–); ^{13}**C NMR** (DMSO-d$_6$, 150 MHz) δ [ppm]: 38.27 (–CH$_2$NH–, –C'H'$_2$NH'–); 66.26 (–OCH$_2$–, –OC'H'$_2$–); 62.56, 66.26, 68.19, 69.43, 72.01, 81.39 (C-2, C-2', C-3, C-3', C-4, C-4', C-5, C-5', C-6, C-6'); 94.94 (C-1, C-1'); 100.71(–OCHO–,–OC'H'O–); 113.88 (C$_c$, C$_c$'); 125.28 (–**C**H=CH$_2$, –**C'**H'=C'H'$_2$); 127.71 (C$_b$, C$_b$'); 130.44 (C$_a$, C$_a$'); 131.56 (–CH=**C**H$_2$, –C'H'=**C'**H'$_2$); 158.59 (C$_d$, C$_d$'); 164.88 (–C(O)–, –C'(O)–). **LR ESI-MS:** m/z calcd for $C_{36}H_{45}O_{13}N$ [M+H]$^+$ 745.3, found 745.5

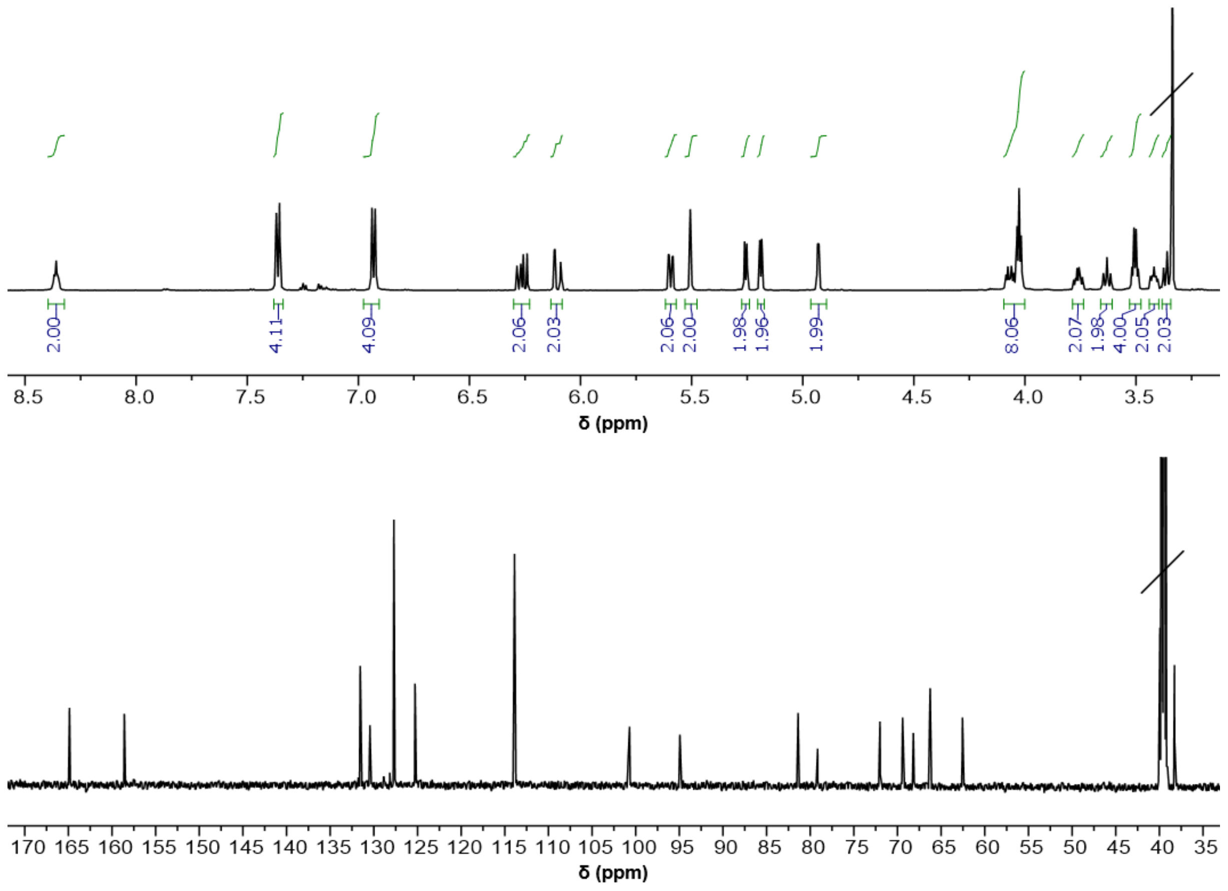

Fig. S4 ^{1}H and ^{13}C NMR spectra of 4,6:4',6'-di-*O*-[*p*-(2-acrylamidoethoxy)benzylidene]-α,α'-D-trehalose **(DT2)**

2.3. Synthetic procedure for MT3 and DT3

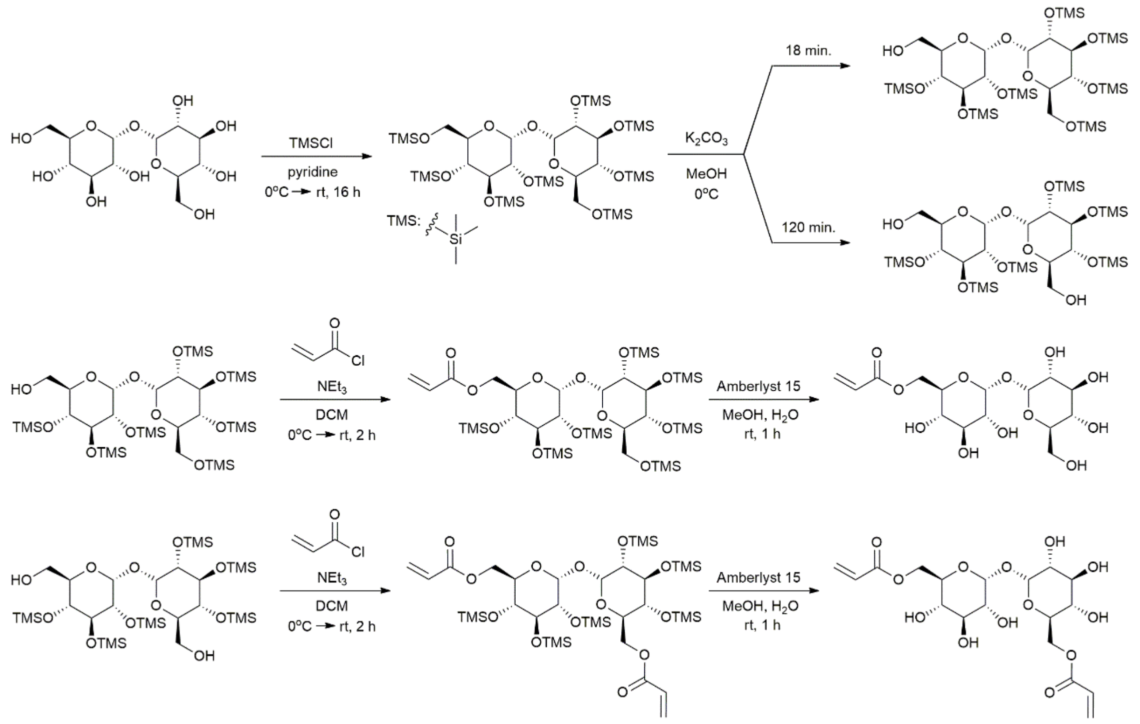

Scheme S3 Synthetic pathway of trehalose monomers **DT3** and **MT3**.

2,3,4,6,2',3',4',6'-okta-*O*-trimethylsilyl-α,α'-D-trehalose

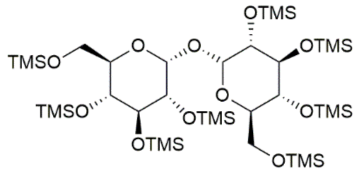

Anhydrous trehalose (21.9 mmol) was dissolved in 80 mL of anhydrous pyridine and the solution was cooled on ice-water bath before trimethylsilyl chloride (362.8 mmol) was added dropwise, while stirring. The reaction was allowed to warm up to room temperature and left overnight. The resulting suspension was diluted with 100 mL of ethyl acetate and extracted with 80 mL of DI water. The organic phase was washed two more times with 80 mL of DI water, dried over $MgSO_4$ and concentrated under vacuum. The residue was evaporated twice with toluene to afford product as white crystals (19.70 g, 98%).

^{1}H NMR ($CDCl_3$, 600 MHz) δ [ppm]: 0.10, 0.12, 0.14, 0.14 (4x s, 72H, –Si(CH_3)$_3$); 3.38 (dd, 2H, J = 9.3, 3.2 Hz, H-2, H-2'); 3.44 (~t, 2H, J = 9.1 Hz, H-4, H-4'); 3.64-3.71 (m, 4H, H-6a, H-6'a, H-6b, H-6'b); 3.76-3.81 (ddd, 2H, J = 9.5, 4.1, 2.3 Hz, H-5, H-5'); 3.89 (~t, 2H, J = 9.0 Hz, H-3, H-3'); 4.91 (d, 2H, J = 3.2 Hz, H-1, H-1'). **^{13}C NMR** ($CDCl_3$, 150 MHz) δ [ppm]: -0.11, 0.32, 1.10, 1.24 (8x –Si(CH_3)$_3$); 62.31 (C-6, C-6'); 71.92, 73.04, 73.41, 73.77 (C-2, C-2', C-3, C-3', C-4, C-4', C-5, C-5'); 94.53(C-1, C-1'). **LR ESI-MS:** m/z calcd for $C_{36}H_{86}O_{11}Si_8Na$ $[M+Na]^+$ 941.4, found 941.7

2,3,4,2',3',4',6'-hepta-*O*-trimethylsilyl-α,α'-D-trehalose

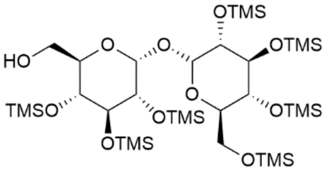

2,3,4,6,2',3',4',6'-okta-*O*-trimethylsilyl-α,α'-D-trehalose (4.3 mmol) was dissolved in 270 mL of methanol and cooled on ice-water bath before K_2CO_3 (4.1 mmol) was added, while intensively stirring. The reaction was quenched after 18 min by transferring into separating funnel containing 200 mL of hexane and extracting twice with 100 mL of DI water. The organic phase was dried over $MgSO_4$ and concentrated under vacuum. Crude product containing starting material, 2,3,4,2',3',4',6'-hepta-*O*-trimethylsilyl-α,α'-D-trehalose and 2,3,4,2',3',4'-hexa-*O*-trimethylsilyl-α,α'-D-trehalose was separated by silica gel flash chromatography (hexane: ethyl acetate 98:2) to afford 2,3,4,2',3',4',6'-hepta-*O*-trimethylsilyl-α,α'-D-trehalose as a colorless oil (1.71 g, 47%). 1.02 g (25%) of starting material was recovered as a first fraction.

^{1}H NMR ($CDCl_3$, 600 MHz) δ [ppm]: 0.10, 0.11, 0.12, 0.14, 0.14, 0.14, 0.16 (7x s, 63H, –$Si(CH_3)_3$); 1.76 (br s, 1H, -OH); 3.38-3.44 (m, 3H, H-2, H-2', H-4'); 3.46 (~t, 2H, J = 9.1 Hz, H-4); 3.63-3.74 (m, 4H, H-6a, H-6b, H-6'a, H-6'b); 3.79 (ddd, 1H, J = 9.5, 4.7 Hz, 2.0 Hz, H-5'); 3.84 (~dt, 1H J = 9.5, 3.5 Hz, H-5); 3.89, 3.92 (2 t, 2H, J = 9.0 Hz, H-3, H-3'); 4.89 (d, 1H J = 3.1 Hz, H-1); 4.94 (d, 1H J = 3.1 Hz, H-1'). **^{13}C NMR** ($CDCl_3$, 150 MHz) δ [ppm]: -0.15, 0.25, 0.27, 1.01, 1.09, 1.17, 1.20 (7x –$Si(CH_3)_3$); 61.88, 62.29 (C-6, C-6'); 71.62, 71.89, 72.87, 72.90, 72.94, 73.54, 73.55, 73.63 (C-2, C-2', C-3, C-3', C-4, C-4', C-5, C-5'); 94.40, 94.57 (C-1, C-1'). **LR ESI-MS:** m/z calcd for $C_{33}H_{78}O_{11}Si_7Na$ $[M+Na]^+$ 869.4, found 869.6

2,3,4,2',3',4'-hexa-*O*-trimethylsilyl-α,α'-D-trehalose

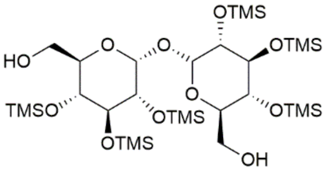

2,3,4,2',3',4'-hexa-*O*-trimethylsilyl-α,α'-D-trehalose was synthetized according to the procedure described for 2,3,4,2',3',4',6'-hepta-*O*-trimethylsilyl-α,α'-D-trehalose, except that alcoholysis was left to proceed for 120 min. Pure product was separated from the crude by silica gel flash chromatography (hexane: ethyl acetate 98:2 → 90:10) as white crystals (2.76 g, 83%).

^{1}H NMR ($CDCl_3$, 600 MHz) δ [ppm]: 0.12, 0.15, 0.16 (3x s, 54H, –$Si(CH_3)_3$); 1.85 (br s, 2H, -OH) 3.42 (dd, 2H, J = 9.3, 3.1 Hz, H-2, H-2'); 3.48 (~t, 2H, J = 9.1 Hz, H-4, H-4'); 3.65-3.74 (m, 4H, H-6a, H-6'a, H-6b, H-6'b); 3.83-3.87 (~td, 2H, J = 9.5, 3.4 Hz, H-5, H-5'); 3.89 (~t, 2H, J = 9.0 Hz, H-3, H-3'); 4.91 (d, 2H, J = 3.1 Hz, H-1, H-1'). **^{13}C NMR** ($CDCl_3$, 150 MHz) δ [ppm]: 0.23, 1.00, 1.15 (6x –$Si(CH_3)_3$); 61.76 (C-6, C-6'); 71.53, 72.91, 73.16,73.49 (C-2, C-2', C-3, C-3', C-4, C-4', C-5, C-5'); 94.80 (C-1, C-1'). **LR ESI-MS:** m/z calcd for $C_{30}H_{70}O_{11}Si_6Na$ $[M+Na]^+$ 797.3, found 797.5

6-*O*-acryloyl-2,3,4,2',3',4',6'-hepta-*O*-trimethylsilyl-α,α'-D-trehalose

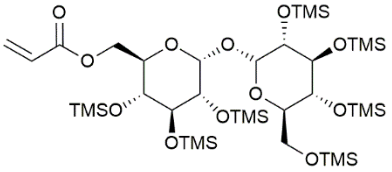

A solution of 2,3,4,2',3',4',6'-hepta-*O*-trimethylsilyl-α,α'-D-trehalose (7.9 mmol) and TEA (17.8 mmol) in 30 mL of anhydrous DCM was cooled on ice water bath, before a solution of acryloyl chloride (11.9 mmol) in 20 mL of anhydrous DCM was added dropwise. The mixture was left to warm up to room temperature. After 2 h the solution was diluted with 160 mL of DCM and extracted twice with 220 mL of brine. The organic phase was dried over $MgSO_4$ and concentrated under vacuum. Crude product was purified by silica gel flash chromatography (hexane: ethyl acetate 98:2) to afford product as colorless oil (5.26 g, 74%).

^{1}H NMR ($CDCl_3$, 600 MHz) δ [ppm]: 0.08, 0.12, 0.13, 0.14, 0.15 (5x s, 63H, –$Si(CH_3)_3$); 3.40 (dd, 1H, J = 9.3, 3.1 Hz, H-2'); 3.42-3.47 (m, 2H, H-2, H-4'); 3.51 (dd, 1H, J = 9.5, 8.6 Hz, H-4); 3.64-3.70 (m, 2H, H-6'a, H-6'b); 3.77 (m, 2H,H-5'); 3.86-3.94 (m, 2H, H-3, H-3'); 4.05 (ddd, 1H J = 9.6, 4.4, 2.4 Hz, H-5); 4.16 (dd, 1H, J = 12.0, 4.4 Hz, H-6a); 4.37 (dd, 1H, J = 12.0, 2.4 Hz, H-6b); 4.90 (d,1H, J = 3.1 Hz, H-1'); 4.96 (d, 1H, J = 3.1 Hz, H-1); 5.84 (dd, 1H, J = 10.4, 1.4 Hz, –CH=C**H**$_2$ *cis*); 6.18 (dd, 1H, J = 17.3, 10.4 Hz, –C**H**=CH_2); 6.44 (dd, 1H, J = 17.3, 1.4 Hz, –CH=C**H**$_2$ *trans*). **^{13}C NMR** ($CDCl_3$, 150 MHz) δ [ppm]: -0.12, 0.30, 0.35, 1.03, 1.07, 1.21, 1.23 (7x –$Si(CH_3)_3$); 62.09, 63.83 (C-6, C-6'); 70.74, 71.72, 72.14, 72.84, 72.97, 73.55, 73.64, 73.73 (C-2, C-2', C-3, C-3', C-4, C-4', C-5, C-5'); 94.39, 94.70 (C-1, C-1'); 128.31 (–**C**H=CH_2); 131.28 (–CH=**C**H_2); 166.31 (–OC(O)–). **LR ESI-MS:** m/z calcd for $C_{36}H_{80}O_{12}Si_7Na$ $[M+Na]^+$ 923.4, found 923.8

6,6'-di-*O*-acryloyl-2,3,4,2',3',4'-hexa-*O*-trimethylsilyl-α,α'-D-trehalose

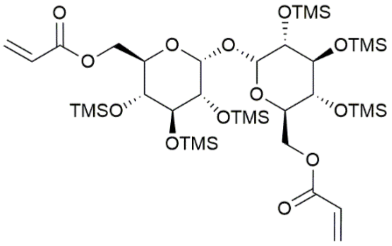

6,6'-di-*O*-acryloyl-2,3,4,2',3',4'-hexa-*O*-trimethylsilyl-α,α'-D-trehalose was synthetized according to the same procedure as described for 6-*O*-acryloyl-2,3,4,2',3',4',6'-hepta-*O*-trimethylsilyl-α,α'-D-trehalose, except that of TEA and acryloyl chloride were used in higher amounts (35.6 and 23.7 mmol respectively). Crude product was purified by silica gel flash chromatography (hexane: ethyl acetate 96:4) to afford the product as white crystals (5.41 g, 78%).

^{1}H NMR ($CDCl_3$, 600 MHz) δ [ppm]: 0.13, 0.14, 0.16 (3x s, 54H, –$Si(CH_3)_3$); 3.46 (dd, 2H, J = 9.3, 3.1 Hz, H-2, H-2'); 3.51 (dd, 2H, J = 9.6, 8.6 Hz, H-4, H-4'); 3.92 (~t, 2H, J = 9.0 Hz, H-3, H-3'); 4.05 (ddd, 2H, J = 9.6, 4.5, 2.4 Hz, H-5, H-5'); 4.16 (dd, 2H, J = 12.0, 4.5 Hz H-6a, H-6'a); 4.36 (dd, 2H, J = 12.0, 2.4 Hz, H-6b, H-6'b); 4.94 (d, 2H, J = 3.1 Hz, H-1, H-1'); 5.85 (dd, 2H, J = 10.4, 1.4 Hz, –CH=C**H**$_2$, –C'H'=C'**H'**$_2$ *cis*);
6.17 (dd, 2H, J = 17.3, 10.4 Hz, –C**H**=CH_2, –C'**H'**=C'H'$_2$); 6.44 (dd, 2H, J = 17.3, 1.4 Hz, –CH=C**H'**$_2$, –C'H'=C'**H'**$_2$ *trans*). **^{13}C NMR** ($CDCl_3$, 150 MHz) δ [ppm]: 0.20, 0.86, 1.06 (6x –$Si(CH_3)_3$); 63.64 (C-6, C-6'); 70.76, 71.96, 72.68, 73.48 (C-2, C-2', C-3, C-3', C-4, C-4', C-5, C-5'); 94.47 (C-1, C-1'), 128.12 (–**C**H=CH_2, –**C**'H'=C'H'$_2$); 131.14 (–CH=**C**H_2, –C'H'=**C**'H'$_2$); 166.10 (–OC(O)–, –OC'(O)–). **LR ESI-MS:** m/z calcd for $C_{36}H_{74}O_{13}$ Si_6Na $[M+Na]^+$ 905.4, found 905.4

6-*O*-acryloyl-α,α'-D-trehalose (MT3)

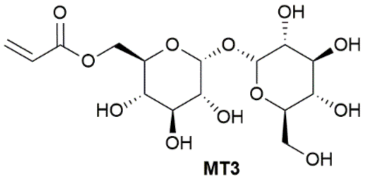

MT3

Amberlyst 15* (8.8 g) was added to the solution of 6-*O*-acryloyl-2,3,4,2',3',4',6'-hepta-*O*-trimethylsilyl-α,α'-D-trehalose (2.5 mmol) in methanol and DI water (330 and 30 mL, respectively) and left at room temperature under stirring for 1 h. Amberlyst 15 was filtered off and the solution was then extracted with 110 mL of hexane. Aqueous phase was separated and concentrated under vacuum at 30 °C to ~15 mL. The residual solution was filtered through the syringe filter (0.045 µm) and freeze dried to afford **MT3** as a white solid (0.97 g, 98%).

*washed several times with methanol

^{1}H NMR (DMSO-d_6, 400 MHz) δ [ppm]: 3.21 – 3.08 (m, 2H, H-4, H-4'); 3.30 – 3.21 (m, 2H, H-2, H-2'); 3.47 (ddd, 1H, J = 11.4, 6.2, 5.0 Hz, H-6'a); 3.60 – 3.50 (m, 3H, H-3, H-3', H-6'b); 3.64 (ddd, 1H, J = 9.9, 4.9, 2.3 Hz, H-5'); 3.96 (ddd, 1H, J = 10.1, 5.6, 2.1 Hz, H-5); 4.16 (dd, 1H, J = 11.8, 5.7 Hz, H-6a); 4.29 (dd, 1H, J = 11.8, 2.1 Hz, H-6b); 4.34 (~t, 1H, J = 5.9 Hz, C-6-OH); 4.67 (d, 1H, J = 6.3 Hz, -OH); 4.69 (d, 1H, J = 6.0 Hz, -OH); 4.74 (d, 1H, J = 4.9 Hz, -OH); 4.76 (d, 1H, J = 5.2 Hz, -OH); 4.84 (d, 1H, J = 3.6 Hz, H-1); 4.85-4.89 (m, 2H, H-1', -OH); 5.09 (d, 1H, J = 5.3 Hz, -OH); 5.94 (dd, 1H J = 10.3, 1.7 Hz, 1H, –CH=C**H**$_2$ *cis*); 6.17 (dd, 1H, J = 17.3, 10.3 Hz, 1H, –C**H**=CH_2); 6.32 (dd, 1H, J = 17.3, 1.7 Hz, 1H, –CH=C**H**$_2$ *trans*). **^{13}C NMR** (DMSO-d_6, 100 MHz) δ [ppm]: 60.73, 63.56 (C-6, C-6'); 69.55, 70.06, 70.10, 71.45, 71.55, 72.59, 72.81 (C-2, C-2', C-3, C-3', C-4, C-4', C-5, C-5'); 93.25, 93.36 (C-1, C-1'); 128.37 (–**C**H=CH_2); 131.48 (–CH=**C**H_2, –C'H'=**C**'H'$_2$); 165.42 (–OC(O)–). **LR ESI-MS:** m/z calcd for $C_{15}H_{24}O_{12}Na$ $[M+Na]^+$ 419.1, found 419.3

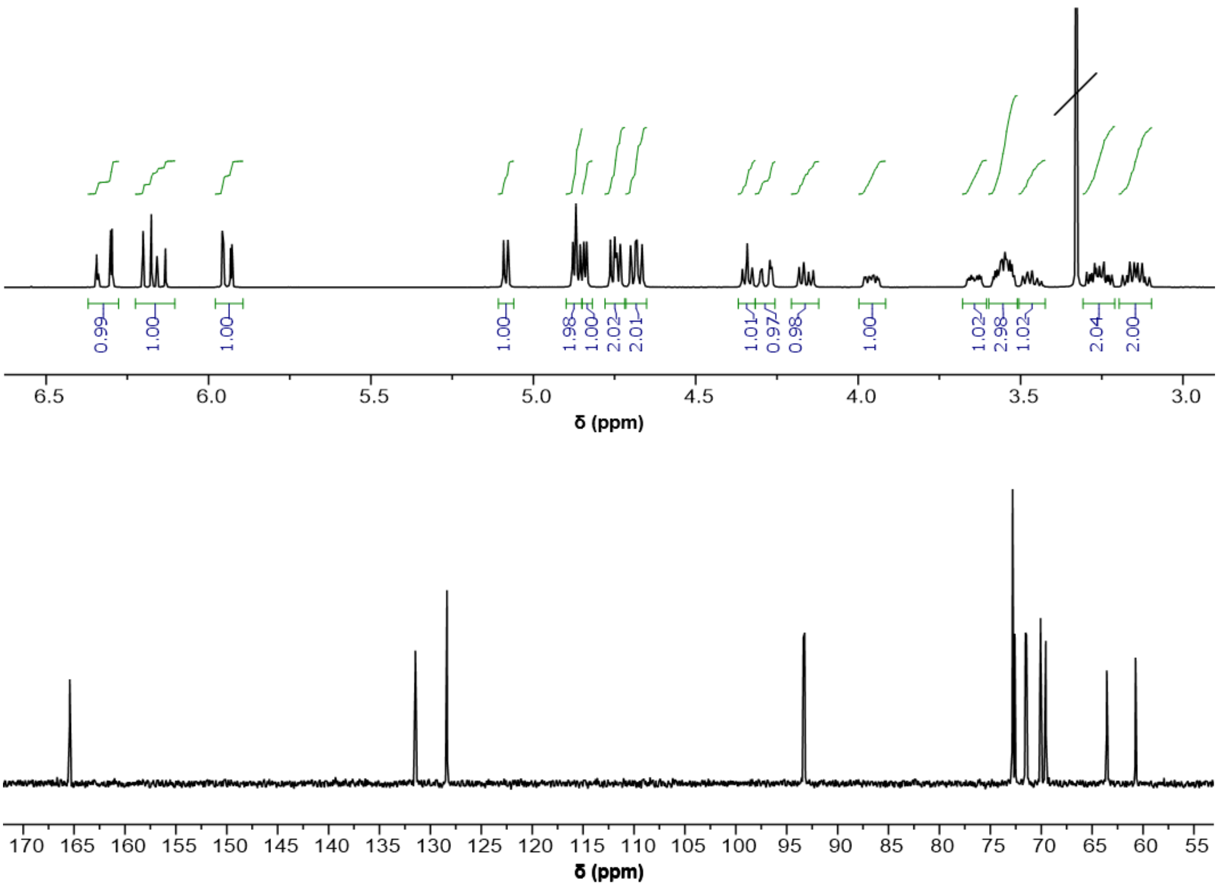

Fig. S5 ^{1}H and ^{13}C NMR spectra of 6-*O*-acryloyl-α,α'-D-trehalose (**MT3**)

6,6'-di-*O*-acryloyl-α,α'-D-trehalose (DT3)

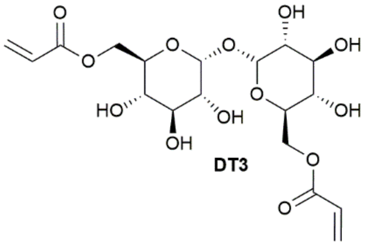

DT3

DT3 was synthetized from 6,6'-di-*O*-acryloyl-2,3,4,2',3',4'-hexa-*O*-trimethylsilyl-α,α'-D-trehalose according to the same procedure as described for **MT3** and obtained as a white solid (1.09 g, 97%)

^{1}H NMR (DMSO-d$_6$, 600 MHz) δ [ppm]: 3.17 (ddd, 2H, J = 10.1, 8.8, 5.4 Hz H-4, H-4'); 3.28 (ddd, 2H, J = 9.7, 6.0, 3.7 Hz, H-2, H-2'); 3.55 (~td, 2H, J = 9.3, 4.9 Hz, H-3, H-3'); 3.97 (ddd, 2H, J = 10.0, 5.5, 2.1 Hz, H-5, H-5'); 4.16 (dd, 2H J = 11.8, 5.5 Hz, H-6a, H-6'a); 4.28 (dd, 2H, J = 11.8, 2.1 Hz, H-6b, H-6'b); 4.80-4.85 (m, 4H, H-1, H-1', 2x -OH); 4.90 (d, 2H J = 5.0 Hz, C-3-OH, C-3'-OH); 5.11 (d, 2H J = 5.4 Hz, C-4-OH, C-4'-OH); 5.94 (dd, 2H J = 10.4, 1.5 Hz, –CH=C**H**$_2$, –C'H'=C'**H'**$_2$ *cis*); 6.18 (dd, 2H, J = 17.3, 10.4 Hz, –C**H**=CH$_2$, –C'**H'**=C'H'$_2$); 6.32 (dd, 2H, J = 17.3, 1.5 Hz, –CH=C**H'**$_2$, –C'H'=C'**H'**$_2$ *trans*). **^{13}C NMR** (DMSO-d$_6$, 150 MHz) δ [ppm]: 63.49 (C-6, C-6'); 69.64, 70.00, 71.38, 72.72 (C-2, C-2', C-3, C-3', C-4, C-4', C-5, C-5'); 93.69 (C-1, C-1'); 128.34 (–**C**H=CH$_2$, –**C'**H'=C'H'$_2$); 131.48 (–CH=**C**H$_2$, –C'H'=**C'**H'$_2$); 165.42 (–OC(O)–, –OC'(O)–). **LR ESI-MS:** m/z calcd for $C_{18}H_{26}O_{13}Na$ $[M+Na]^+$ 473.1, found 473.2

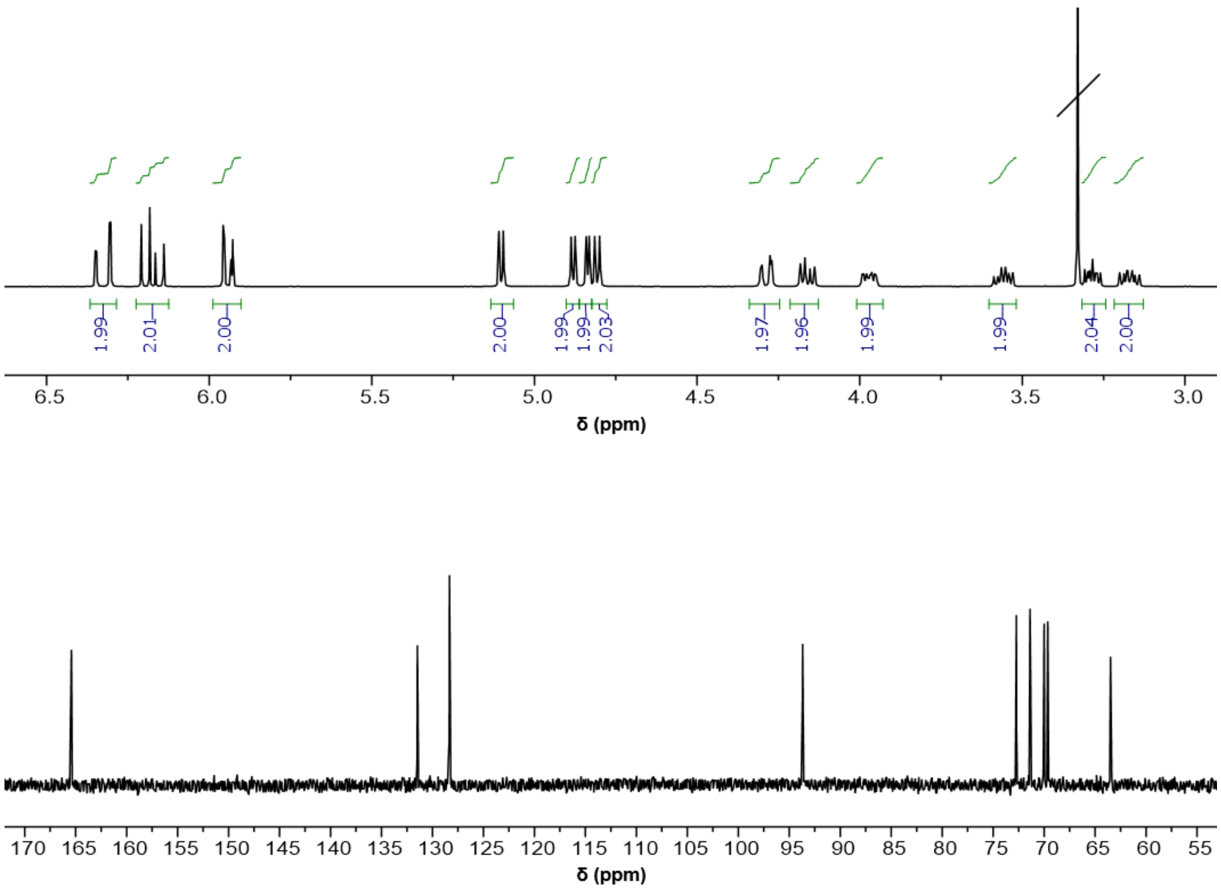

Fig. S6 ^{1}H and ^{13}C NMR spectra of 6,6′-di-*O*-acryloyl-α,α′-D-trehalose **(DT3)**

3. Synthesis and study on hydrogels

3.1. Feed composition for the synthesis of acid-labile hydrogels

Table S1 Monomer feed compositions and hydrogels yields

Sample code	DMAM / **MTx** (w/w)	DMAM [mg] ([mmol])	**MT1** or **MT2** [mg] ([mmol])	**DT1** or **DT2** [mg] ([mmol])	Crosslinker to monomers ratio [mmol/g]	Yield [%]
**DMAM-MT1-0**	100/0	140 (1.412)	-	15 (0.011)	0.08	88
**DMAM-MT1-15**	85/15	119 (1.200)	21 (0.024)	15 (0.011)	0.08	83
**DMAM-MT1-30**	70/30	98 (0.989)	42 (0.048)	15 (0.011)	0.08	81
**DMAM-MT2-0**	100/0	140 (1.412)	-	7.9 (0.011)	0.08	88
**DMAM-MT2-15**	85/15	119 (1.200)	21 (0.039)	7.9 (0.011)	0.08	85
**DMAM-MT2-30**	70/30	98 (0.989)	42 (0.077)	7.9 (0.011)	0.08	80

3.2. Feed composition for the synthesis of alkali-labile hydrogels

Table S2 Monomer feed compositions and hydrogels yields

Sample code	AM / **MT3** (w/w)	AM [mg] ([mmol])	**MT3** [mg] ([mmol])	**DT3** [mg] ([mmol])	Crosslinker to monomers ratio [mmol/g]	Yield [%]
**AM-MT3-0**	100/0	108 (1.519)	-	8.7 (0.019)	0.18	96
**AM-MT3-25**	75/25	81 (1.140)	27 (0.068)	8.7 (0.019)	0.18	96
**AM-MT3-50**	50/50	54 (0.760)	54 (0.136)	8.7 (0.019)	0.18	91
**AM-MT3-75**	25/75	27 (0.380)	81 (0.204)	8.7 (0.019)	0.18	87
**AM-MT3-50-1.5**	50/50	81 (1.140)	81 (0.204)	8.7 (0.019)	0.12	84

3.3. Estimation of trehalose content originating from mono- and diacetal

Wt% of trehalose originating from diacetal ($wt\%_{TRE(DT)}$) was estimated based on the enzymatically determined content of trehalose ($wt\%_{T=\Sigma TRE}$) and mole fraction of diacetal (X_{DT}). X_{DT} was calculated from the integration of trehalose anomeric signal ($I_{TRE\ H\text{-}1,H\text{-}1'}$) and aldehyde aromatic signals ($I_{Ar(A)}$, $I_{Ar(B)}$) in ^1H NMR spectra of degradation products (Fig. S1-S4). The following equation was used:

$$\mathrm{wt\%_{TRE(DT)}} = \mathrm{X_{DT}} * \mathrm{wt\%_{\Sigma TRE}} = \frac{\frac{\mathrm{I_{Ar(A)}} + \mathrm{I_{Ar(B)}}}{2}}{\mathrm{I_{TRE\ H-1,H-1'}}} * \mathrm{wt\%_{\Sigma TRE}}$$

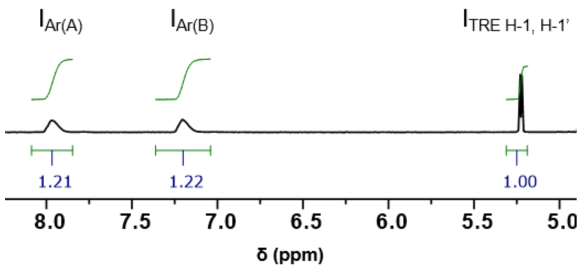

Fig. S7 Signals in ^{1}H NMR spectra of degradation products of acid-labile hydrogels used for estimation of trehalose content originating from mono- and diacetal.

Table S3 Estimation of trehalose content originating from mono- and diacetal in acid-labile hydrogels

Sample code	$wt\%_{TRE}$	X_{DT}	$X_{MT} = 1 - X_{DT}$	$wt\%_{TRE(DT)}$	$wt\%_{TRE(MT)}$
DMAM-MT1-0	1.7	1.00	-	1.7	-
DMAM-MT1-15	6.1	0.35	0.65	2.1	4.0
DMAM-MT1-30	11.4	0.22	0.78	2.5	8.9
DMAM-MT2-0	2.0	1.00	-	2.0	-
DMAM-MT2-15	8.5	0.27	0.73	2.3	6.2
DMAM-MT2-30	16.4	0.17	0.83	2.8	13.6

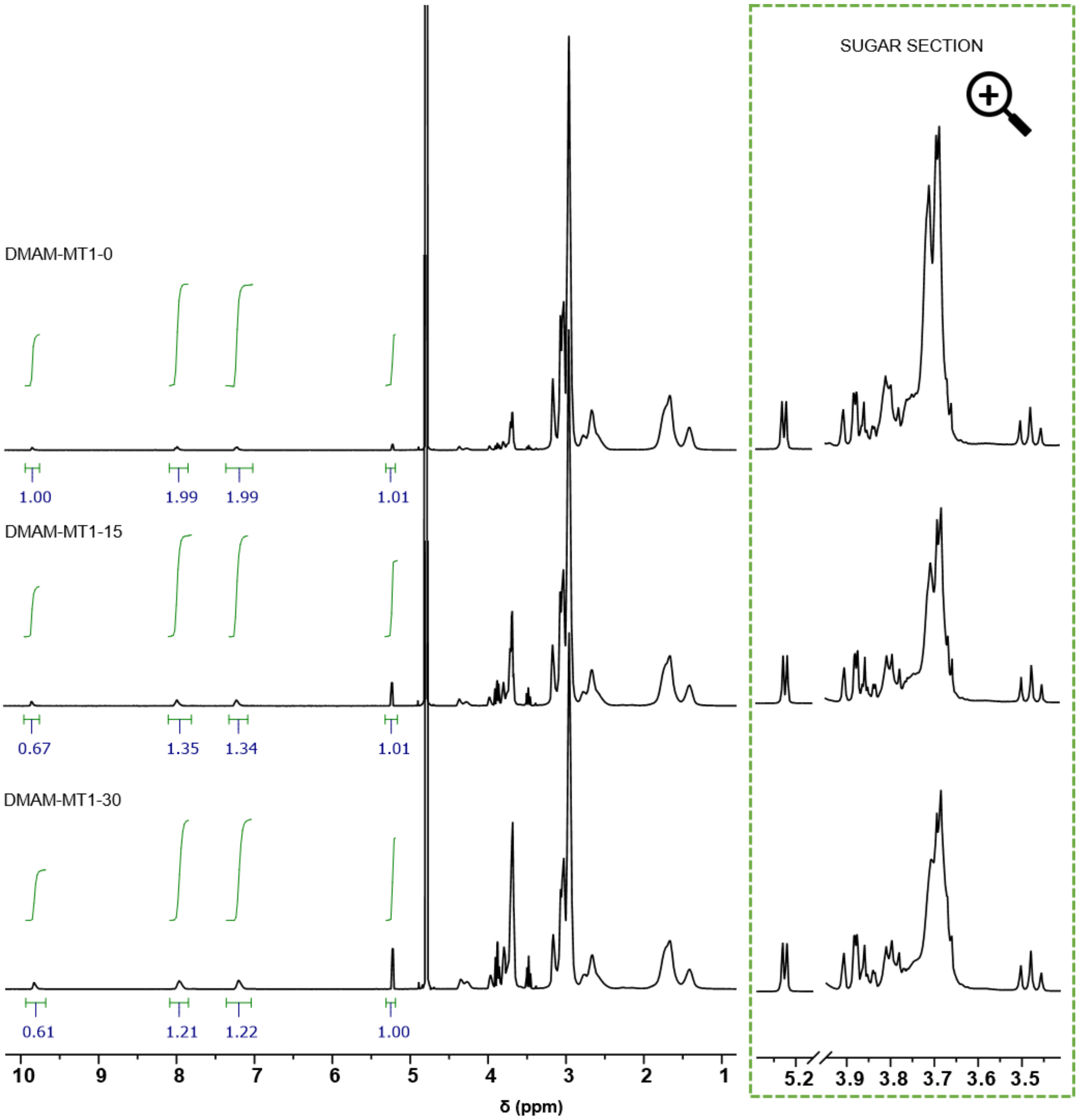

Fig. S8 ^{1}H NMR spectra of degradation products of acid-labile hydrogels containing trehalose monomers set MT1/DT1

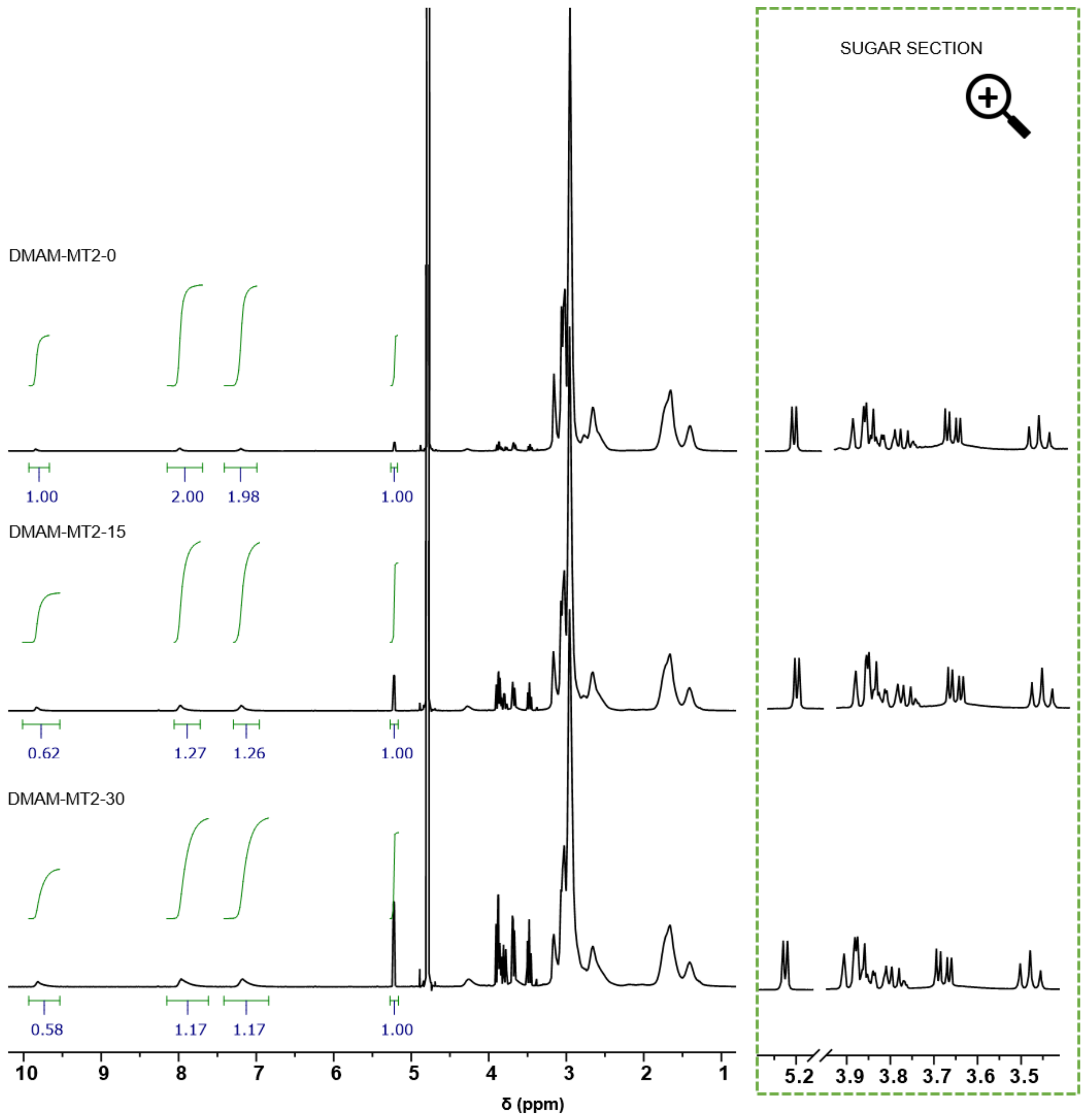

Fig. S9 ^{1}H NMR spectra of degradation products of acid-labile hydrogels containing trehalose monomers set MT2/DT2

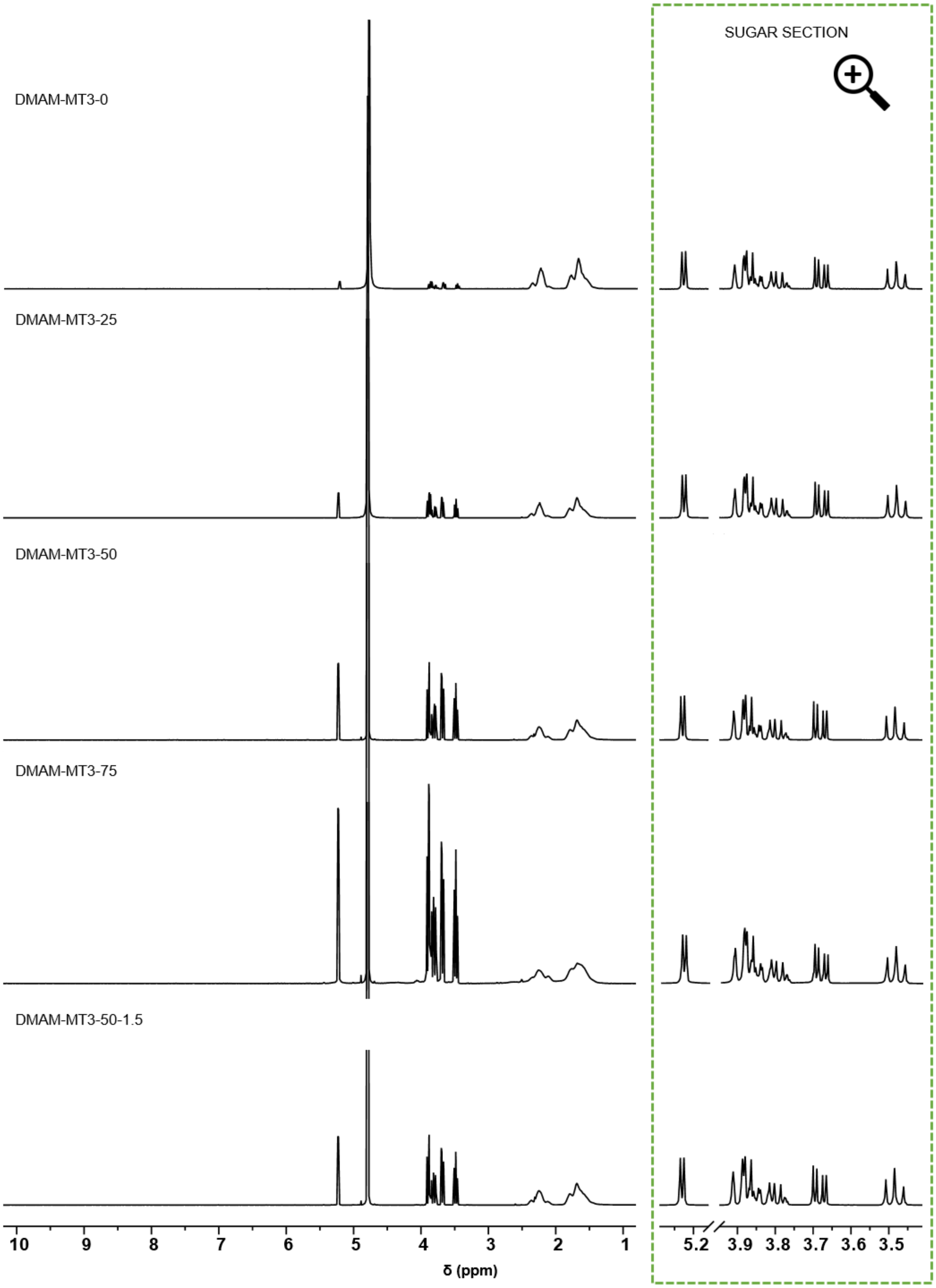

Fig. S10 ^{1}H NMR spectra of degradation products of alkali-labile hydrogels containing trehalose monomers set MT3/DT3

4. References

[1] R. Søndergaard, S. Strobel, E. Bundgaard, K. Norrman, A.G. Hansen, E. Albert, G. Csaba, P. Lugli, M. Tornow, F.C. Krebs, *J. Mater. Chem.* 19 (**2009**) 3899.

[2] R. Mahou, C. Wandrey, *Polymers* 4 (**2012**) 561.

[3] H. Li, P. Zhang, L.P. Smaga, R.A. Hoffman, J. Chan, *J. Am. Chem. Soc.* 137 (**2015**) 15628.

[4] N. Hamada, K. Kazahaya, H. Shimizu, T. Sato, Synlett 6 (**2004**) 1074.
